# Phosphorylation of human phospholipase A1 DDHD1 at newly identified phosphosites affects its subcellular localization

**DOI:** 10.1016/j.jbc.2021.100851

**Published:** 2021-06-03

**Authors:** Naoki Matsumoto, Yoko Nemoto-Sasaki, Saori Oka, Seisuke Arai, Ikuo Wada, Atsushi Yamashita

**Affiliations:** 1 Faculty of Pharma-Science, Teikyo University, Itabashi-Ku, Tokyo, Japan; 2 Department of Cell Science, Institute of Biomedical Sciences, Fukushima Medical University School of Medicine, Fukushima City, Fukushima, Japan

**Keywords:** phospholipase A1 (PLA1), DDHD domain containing 1 (DDHD1), hereditary spastic paraplegia (HSP), phosphorylation, Phos-tag, cyclin-dependent kinase (CDK), λPP, lambda protein phosphatase, CDK, cyclin-dependent kinase, CK2, casein kinase 2, GSK-3, glycogen synthase kinase-3, HSP, hereditary spastic paraplegia, IB, immunoblot, IMAC, immobilized metal affinity chromatography, IP, immunoprecipitation, LPA, lysophosphatidic acid, LPI, lysophosphatidylinositol, MALDI-TOF MS, matrix-assisted laser desorption ionization time-of-flight mass spectrometry, PA, phosphatidic acid

## Abstract

Phospholipase A1 (PLA1) hydrolyzes the fatty acids of glycerophospholipids, which are structural components of the cellular membrane. Genetic mutations in DDHD1, an intracellular PLA1, result in hereditary spastic paraplegia (HSP) in humans. However, the regulation of DDHD1 activity has not yet been elucidated in detail. In the present study, we examined the phosphorylation of DDHD1 and identified the responsible protein kinases. We performed MALDI-TOF MS/MS analysis and Phos-tag SDS-PAGE in alanine-substitution mutants in HEK293 cells and revealed multiple phosphorylation sites in human DDHD1, primarily Ser8, Ser11, Ser723, and Ser727. The treatment of cells with a protein phosphatase inhibitor induced the hyperphosphorylation of DDHD1, suggesting that multisite phosphorylation occurred not only at these major, but also at minor sites. Site-specific kinase-substrate prediction algorithms and *in vitro* kinase analyses indicated that cyclin-dependent kinase CDK1/cyclin A2 phosphorylated Ser8, Ser11, and Ser727 in DDHD1 with a preference for Ser11 and that CDK5/p35 also phosphorylated Ser11 and Ser727 with a preference for Ser11. In addition, casein kinase CK2α1 was found to phosphorylate Ser104, although this was not a major phosphorylation site in cultivated HEK293 cells. The evaluation of the effects of phosphorylation revealed that the phosphorylation mimic mutants S11/727E exhibit only 20% reduction in PLA1 activity. However, the phosphorylation mimics were mainly localized to focal adhesions, whereas the phosphorylation-resistant mutants S11/727A were not. This suggested that phosphorylation alters the subcellular localization of DDHD1 without greatly affecting its PLA1 activity.

Phospholipase A1 (PLA1) is an enzyme that catalyzes the hydrolysis of the *sn*-1 fatty acids of glycerophospholipids, which are structural components of the cellular membrane (1, 2, 3, 4, 5). The formation of 2-acyl lysophospholipid mediators and the clearance of bioactive lipids, such as phosphatidic acid (PA), have been suggested as physiological functions; however, the underlying mechanisms remain unclear. Although PLA1 activity has been detected in many tissues in various organisms, the number of PLA1 enzymes is smaller than that of phospholipase A2. PLA1 enzymes are classified as extracellular and intracellular proteins (6). In mammals, extracellular PLA1s comprise phosphatidylserine-specific PLA1 (PS-PLA1) (7) and membrane-associated phosphatidic-acid-specific PLA1 (mPA-PLA1α and mPA-PLA1β) (8, 9). In contrast, intracellular PLA1s comprise DDHD domain containing 1 (DDHD1) (2, 3, 10, 11, 12) and its homologs DDHD2/KIAA0725p (13) and p125/Sec23-interacting protein (14). DDHD-type PLA1 family enzymes possess a conserved consensus sequence of lipases (serine esterases), but do not exhibit significant sequence homology to other phospholipases. Although DDHD-type PLA1 family enzymes contain the DDHD domain, which was first identified as a long stretch of amino acids in the C-terminal region of the N-terminal domain interacting-receptor/*Drosophila* retinal degeneration B (Nir/rdgB) proteins with four conserved amino acid residues (Asp, Asp, His, and Asp; DDHD), the physiological functions and roles of the domain have not yet been elucidated in detail (15). DDHD1 was previously identified as PA-preferring PLA1 (PA-PLA1) (2, 3, 10, 11, 12). Although the PLA1 activity of DDHD2 has been confirmed, that of p125 remains unknown.

Distinct from secreted/extracellular PA-PLA1 isoforms that function in outside of the plasma membrane, DDHD-type PLA1 enzymes locate in the cytoplasm (4, 5, 6).

Several mutations in the human DDHD1 gene have recently been reported in patients with autosomal-recessive forms of hereditary spastic paraplegia (HSP), termed *SPG28* (16, 17, 18, 19, 20). DDHD2 gene mutations have also been detected in patients with HSP, termed the *SPG54* subtype (21). HSP is a clinically and genetically heterogeneous group of inherited neurodegenerative disorders that are characterized by slowly progressive lower-limb spasticity due to degeneration of the corticospinal tract (22). Mitochondrial dysfunction has been strongly implicated in the pathogenesis of many neurodegenerative disorders, including Alzheimer’s disease, Parkinson’s disease, Huntington’s disease, and amyotrophic lateral sclerosis (23). Recent studies suggested that DDHD1 regulates mitochondrial dynamics *via* the hydrolysis of PA (24). We previously reported that the DDHD1 mutations found in HSP patients altered the mitochondrial architecture and bioenergetics with increased oxidative stress in human lymphoblasts (16). We also proposed the involvement of DDHD1 in the generation of 2-arachidonoyl lysophosphatidylinositol (LPI), which is a natural ligand for the novel cannabinoid receptor GPR55 (4, 16, 25, 26, 27); however, the relationship between HSP (*SPG28* subtype) and LPI/GPR55 has not yet been elucidated and the underlying pathogenic mechanisms remain unclear.

Posttranslational modifications play a key role in many cellular processes such as signaling and regulatory processes (28). Protein phosphorylation is the most common posttranslational modification and is involved in intracellular signaling through the regulation of individual enzymes. However, limited information is currently available on the posttranslational modifications and basal regulatory mechanisms of the PLA1 activity of human DDHD1. In addition to DDHD1 gene mutations, dysregulation of the PLA1 activity of DDHD1 may also correlate with the development of pathogenesis in patients with neurodegenerative disorders.

In the present study, we examined an important posttranslational modification, the “phosphorylation” of human DDHD1. We identified multisite phosphorylated serine and threonine residues using matrix-assisted laser desorption ionization time-of-flight mass spectrometry (MALDI-TOF MS), Phos-tag SDS-PAGE, and systematic alanine-substituted mutagenesis (Fig. S1). In addition, we demonstrated that the potential responsible protein kinases are cyclin-dependent kinases (CDKs) for the major phosphorylation sites Ser11 and Ser727. We also found that phosphorylation alters the subcellular localization of DDHD1 without large change of PLA1 activity.

## Results

### Phosphorylation of human DDHD1

Based on the primary structure of human DDHD1, multiple phosphorylation sites were predicted by various software programs, such as NetPhos 3.1 Server (http://www.cbs.dtu.dk/services/NetPhos/), and analyses revealed multiple phosphorylation sites in human DDHD1. Phosphoserines, S8, S11, S104, S130, S139, S332, S723, S727, S738, and S806 are predicted with a high score (over 0.500) (Table S1). CDK5 and glycogen synthase kinase-3 (GSK3) were predicted to be involved in most of the phosphorylation sites, whereas casein kinase 1 (CK1) was predicted to be involved at S130 and S738. To confirm these predictions, we examined the phosphorylation of human DDHD by Zn^2+^-Phos-tag SDS-PAGE, which enables the separation of phosphorylated proteins based on their slower mobility relative to their unphosphorylated counterparts in gels containing a phosphate-binding tag (29).

**Figure 1:**
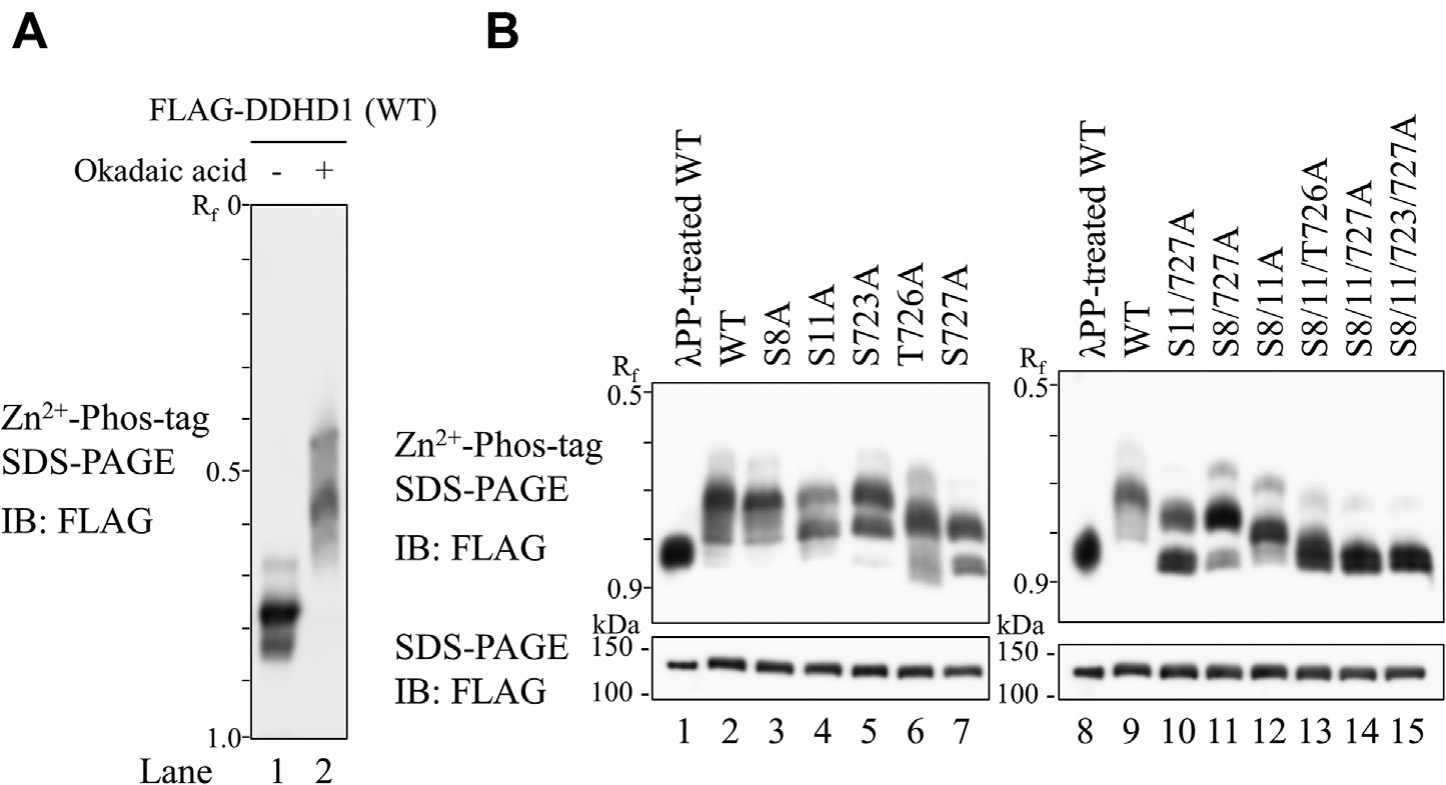
**Human DDHD1 is a phosphorylated protein.** Wild-type FLAG-DDHD1 (WT) and single (S8A, S11A, S723A, T726A, and S727A) or multiple (S8/11A, S11/727, S11/727A, S8/11/T726A, S8/11/727A, and S8/11/723/727A) Ser (Thr)-to-Ala substitution mutants were expressed in HEK293 cells. *A*, electrophoretic mobility shift in WT FLAG-DDHD1 by the inhibition of protein phosphatases. WT FLAG-DDHD1-expressing HEK293 cells were cultured in the absence (lane 1) or presence (lane 2) of 1 μM okadaic acid for 4 h in growth medium containing 10% FBS. Cell extracts were analyzed by Zn^2+^ Phos-tag SDS-PAGE following immunoblot analysis (IB). *B*, electrophoretic mobility shift in WT and Ala substitution mutants of human DDHD1 by phosphorylation. WT (lanes 1, 2, 8, and 9) and Ala substitution mutant (lanes 3–7 and 10–15)-expressing HEK293 cells were cultured in growth medium containing 10% FBS. Cell extracts were analyzed by Zn^2+^ Phos-tag SDS-PAGE (*upper panels*) or normal SDS-PAGE (*lower panels*) following immunoblot analysis (IB). In some cases, cell extracts from WT-expressing HEK293 cells were treated with λPP (lanes 1 and 8) to confirm electrophoretic mobility by phosphorylation. The R_f_ value of 1.0 is defined as the position of bromphenol blue dye. Results are from one experiment representative of three independent experiments.

After FLAG-tagged human DDHD1 was expressed in HEK293 cells, cells were treated with okadaic acid, an inhibitor of protein phosphatases 1 and 2A in growth medium containing 10% fetal bovine serum (FBS). As shown in Figure 1*A*, the inhibition of endogenous phosphatase activity with okadaic acid altered the mobility of DDHD1 in Phos-tag SDS-PAGE followed by immunoblot analysis; many slower bands were detected by the anti-FLAG M2 antibody. In addition, even when cells were cultivated in normal growth medium with 10% FBS and 25 mM glucose, DDHD1 was phosphorylated because treatment with lambda protein phosphatase (λPP) increased mobility (Fig. 1*B*, lane 1 *versus* 2, lane 8 *versus* 9). Therefore, human DDHD1 was suggested to be highly phosphorylated and may be reversibly regulated in phosphorylation–dephosphorylation states by protein kinases and phosphatases.

### Identification of DDHD1 phosphorylation sites by MALDI-TOF MS/MS

We attempted to identify phosphorylation sites in human DDHD1 using MALDI-TOF MS/MS analysis. FLAG-DDHD1-expressing HEK293 or PANC1 cells were treated with okadaic acid, and FLAG-DDHD1 was purified by immunoprecipitation and SDS-PAGE. After the in-gel digestion of purified FLAG-DDHD1 with trypsin or Asp-N, the resultant phosphopeptides were further purified by immobilized metal affinity chromatography (IMAC) or titanium dioxide (TiO_2_), zirconium dioxide (ZrO_2_), or TiO_2_/ZrO_2_ affinity chromatography. Peptides were analyzed by MALDI-TOF MS/MS.

**Figure 2:**
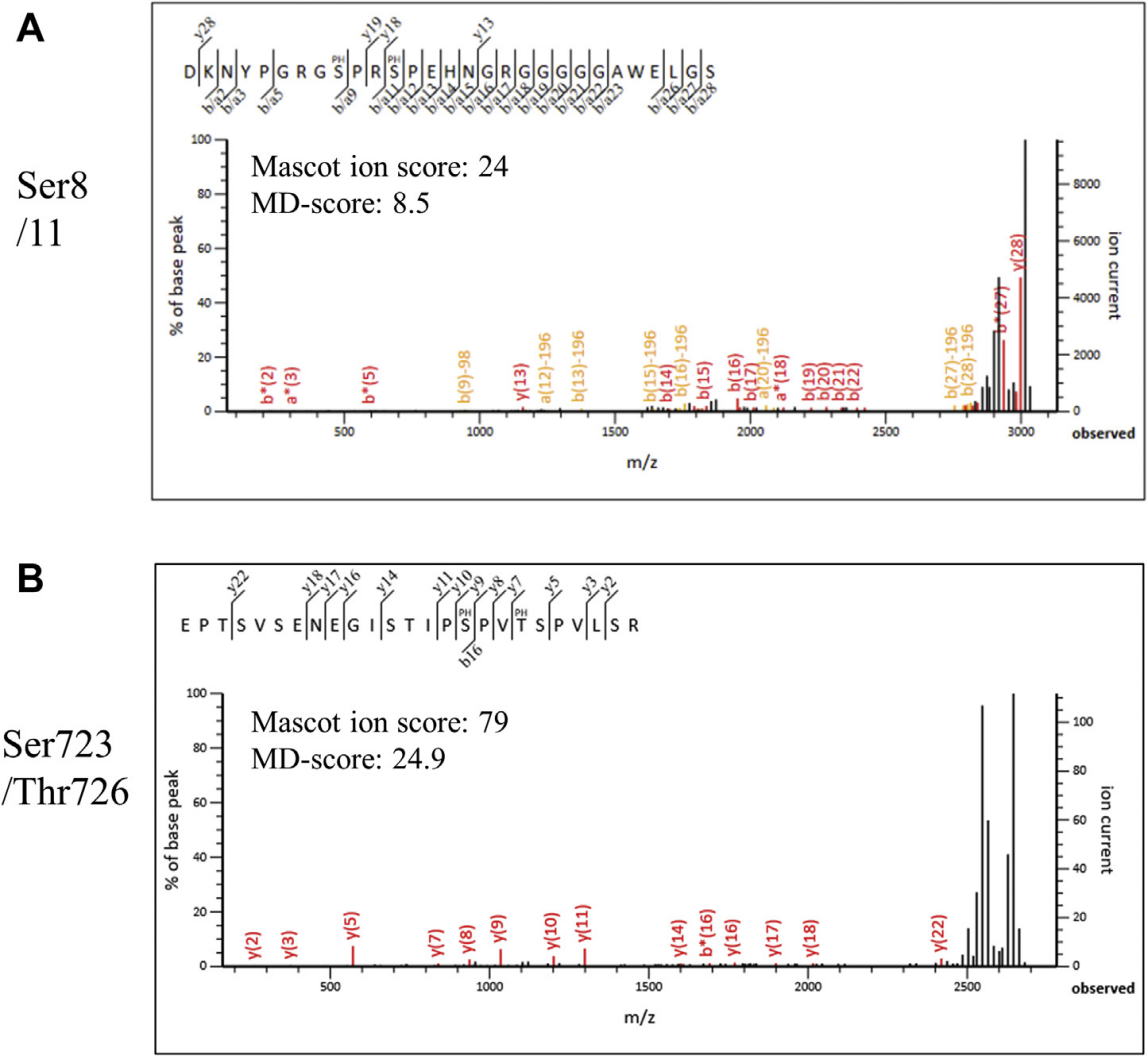
**Identification of phosphorylated sites in DDHD1 with or without okadaic acid.** Extracts from FLAG-DDHD1-expressing and okadaic-acid-treated cells were immunoprecipitated with anti-DYKDDDDK (FLAG) antibody, fractionated by SDS-PAGE, and subjected to in-gel digestion with trypsin or AspN. The resultant peptides were analyzed by MALDI-TOF MS/MS. MS/MS spectra of a diphosphorylated peptide at Ser8 and Ser11 (*A*) and those at Ser723 and Thr726 (*B*) were depicted as representatives of the peptides shown in Table 1. The sequences of identified peptides are shown above the mass spectra; phosphorylation sites are indicated by *PH* on amino acids; *bn* denotes the ion generated by the cleavage of the peptide bond after the *n*th amino acid from the amino terminus; *yn* denotes the ions generated from the carboxyl terminus.

The Mascot analysis using the database of the human SwissProt 2016_07 (551,705 sequences), 2016_08 (551,987 sequences), or 2017_04 (554,241 sequences), or FLAG-tagged human DDHD1 sequence revealed more than ten phosphopeptides of DDHD1 with significant scores (Table 1). These phosphorylation sites account for 11% (nine serines) and 2% (one threonines) of the total because human DDHD1 protein contains 82 serines and 50 threonines. Most phosphoserines or phosphothreonine, except S727, which were predicted by NetPhos 3.1 (Table S1), were detected by MALDI-TOF MS/MS (Table 1). Phosphothreonine 726 was detected by MS/MS, although the score of NetPhos 3.1 for threonine 726 was not high (0.448). The prediction also suggested that GSK3 is involved in the phosphorylation of threonine 726. Two representative MS/MS fragment spectra of DDHD1-derived phosphopeptides are shown in Figure 2 (the spectra of other peptides are shown in Fig. S2). The peptide DK^2^NY…GS^28^ was phosphorylated at serine 8 (Ser8) and Ser11 (Fig. 2*A*). On the other hand, the peptide ^708^EP…SR^732^, containing six serines and three threonines, was phosphorylated at Ser723 and threonine 726 (Thr726) (Fig. 2*B*). Single-phosphorylated peptides at only Ser8, Ser11, or Ser723 were also detected, but not at Thr726 (Table 1, Fig. S2, *A*, *B*, and *G*). The phosphorylation of DDHD1 at Ser8, Ser11, and Ser723 was also predicted using a protein phosphorylation site predictor; however, the software implied a greater potential for phosphorylation at Ser727 than at Thr726 (Table S1). Therefore, we presumed that Ser727 was also a candidate residue for the phosphorylation of DDHD1. Evidence supporting the phosphorylation of Ser727 is described later (Fig. 1*B*). We considered the phosphorylation of Ser727 to be sensitive to dephosphorylation by phosphatase or the detection process of MALDI-TOF MS/MS. Alternately, we considered the possibility that Ser727 cannot be phosphorylated when Thr726 is already phosphorylated.

**Table 1:** Peptides with phosphorylation sites found in human DDHD1

Theoretical DDHD1 phosphorylation sites	Start	End	Sequence	Mascot ion score	Expectation value	MD-score	m/z ratio	Charge	Source
Ser8	2	28	DKNYPGRGpSPRSPEHNGRGGGGGAWELGS	44	4.3 × 10^−5^	8.3	3033.0424	1+	HEK293, Asp-N, ZrO_2_/TiO_2_
Ser11	2	28	DKNYPGRGSPRpSPEHNGRGGGGGAWELGS	60	9.6 × 10^−7^	10	3033.0424	1+	HEK293, Asp-N, ZrO_2_/TiO_2_
Ser8, Ser11	2	28	DKNYPGRGpSPRpSPEHNGRGGGGGAWELGS	24	3.6 × 10^−3^	8.5	3113.0223	1+	HEK293, Asp-N, ZrO_2_/TiO_2_
Ser104, Ser139	102	143	YYpSEGESGGGGSSLSLHPPQQPPLVPTNSGGGGATGGpSPGER	71	7.4 × 10^−6^	28.1	4144.0871	1+	PANC1, Okadaic acid, Trypsin, TiO_2_
Ser130	102	143	YYSEGESGGGGSSLSLHPPQQPPLVPTNpSGGGGATGGSPGER	141	6.1 × 10^−13^	11.5	4064.1072	1+	HEK293, Trypsin, TiO_2_
Ser139	102	143	YYSEGESGGGGSSLSLHPPQQPPLVPTNSGGGGATGGpSPGER	88	1.2 × 10^−7^	5.2	4064.1072	1+	HEK293, Trypsin, TiO_2_
Ser332	318	336	GQQMQENFDIEVSKpSIDGK	41	1.1 × 10^−2^	12	2233.3063	1+	PANC1, Okadaic acid, Trypsin, TiO_2_
Ser723	708	732	EPTSVSENEGISTIPpSPVTSPVLSR	53	7.7 × 10^−4^	10.7	2663.7792	1+	HEK293, Okadaic acid, Trypsin, ZrO_2_/TiO_2_
Ser723, Thr726	708	732	EPTSVSENEGISTIPpSPVpTSPVLSR	79	2 × 10^−6^	24.9	2743.7591	1+	HEK293, Trypsin, ZrO_2_/TiO_2_
Ser738	733	744	RHYGEpSITNIGK	21	7.6 × 10^−3^	10.6	1454.4813	1+	PANC1, Okadaic acid, Trypsin, TiO_2_
Ser806	783	809	DEKKPVASPSATTVGTQTLPHSSpSGFL	94	2.2 × 10^−8^	9	2822.9671	1+	HEK293, Okadaic acid, Asp-N, ZrO_2_/TiO_2_

Underlined sequences are derived from the FLAG epitope tag at the N terminus of DDHD1.

### Identification of DDHD1 phosphorylation sites by site-directed mutagenesis and Phos-tag SDS-PAGE

To confirm the phosphorylation sites of DDHD1, we created several site-directed mutants in which phosphorylation sites identified by MALDI-TOF MS/MS (Table 1) were changed to alanine (Fig. S1). The substitutions to alanine resulted in the creation of phosphorylation-resistant forms of DDHD1. These constructs were transiently transfected into HEK293 cells, and changes in the mobility of DDHD1 mutants from that of wild-type DDHD1 (WT) were analyzed by Phos-tag SDS-PAGE. The WT enzyme and serine or threonine to alanine substitution enzymes had several bands that migrated slower than those of λPP-treated WT, suggesting that WT and the mutant enzymes were phosphorylated (Fig. 1*B*). With the mutant enzymes, S11A (the mutant of serine 11 to alanine), S723A, T726A, and S727A, there were faster migrating bands and fewer slower bands than with WT. This demonstrated that the amino acid residues Ser11, Ser723, and Thr726 were phosphorylated and confirmed the results of MALDI-TOF MS/MS. Although the phosphorylation of Ser727 was not observed by MALDI-TOF MS/MS, Phos-tag SDS-PAGE of the S727A mutant revealed its phosphorylation.

Phos-tag SDS-PAGE of DDHD1 with multiple mutations revealed that its migration was generally faster than that of WT and the single and double/triple mutants (Fig. 1*B*). These results again indicated that Ser8, Ser11, Ser723, Thr726, and Ser727 were all phosphorylated. The phosphorylation of Ser8 is easily detectable based on comparison of the mobility of S11/727A (Fig. 1*B*, lane 10) and S8/11/727A (Fig. 1*B*, lane 14). The mobility of the bands of the S8/11/727A mutant was similar to that of the bands of the λPP-treated WT enzyme (Fig. 1*B*, lanes 8 and 14); however, doublet bands were observed with the S8/11/727A mutant. In addition, the mobility of the bands of the S8/11/727A mutant was similar to that of S8/11/723/727A (Fig. 1*B*, lanes 14 and 15), suggesting that the degree of phosphorylation at Ser723 was low. Therefore, Ser8, Ser11, and Ser727 are the major phosphorylation sites of DDHD1 in HEK293 cells.

The fast migrating band was broader in the Phos-tag SDS-PAGE of S8/11/T726A (Fig. 1*B*, lane 13). Comparison with S8/11A (Fig. 1*B*, lane 12) revealed that the degree of phosphorylation at Thr726 is high because migration was increased by mutation at Thr726. In contrast, the fast migrating band of S8/11/T726A was broader upward than that of S8/11/727A (Fig. 1*B*, lane 14), suggesting a greater degree of phosphorylation at Ser727 than at Thr726, whereas Ser8 and Ser11 were not phosphorylated.

The inhibition of phosphorylation at Thr726 may suppress that of other serine, threonine, and tyrosine residues (or the activation of dephosphorylation) because very fast migrating bands were observed in the Phos-tag SDS-PAGE of the T726A mutant (Fig. 1*B*, lane 6). As the front of the broader bands migrated faster than that of λPP-treated WT, the mutation at T726 may affect not only the inhibition of phosphorylation, but also conformational changes in the 3D structure of DDHD1.

However, detailed comparisons suggested that the degree of phosphorylation differed at each serine or threonine. The phosphorylation of Ser8 was not prominent because the mobility of S8A was not as affected as that of WT (Fig. 1*B*, lanes 2 and 3). However, based on comparison of the mobility of S727A (Fig. 1*B*, lane 7) and S8/727A (Fig. 1*B*, lane 11), the intensity of the fast migrating band decreased, whereas that of slower migrating ones, which were (hyper-) phosphorylated, markedly increased when Ser8 and Ser727 were not phosphorylated. Thus, the phosphorylation of Ser8 affected the phosphorylation of other sites. In contrast, there were more fast migrating bands and fewer slower bands in S8/11A (Fig. 1*B*, lane 12) than in S11A (Fig. 1*B*, lane 4); however, very slow migrating bands, which were (hyper-) phosphorylated, were also observed. These results again suggested that phosphorylation is increased when Ser8 and Ser11 are not phosphorylated.

Similar results were observed in analyses employing other human cell lines such as HeLa, PANC1, and HepG2 cells (Fig. S3). Collectively, the main phosphorylation sites of human DDHD1 were Ser8, Ser11, Ser723, and Ser727 when these cells were cultivated in growth medium. However, hyperphosphorylated bands were observed when S8/11/723/727A mutant-expressing HEK293 cells were treated with okadaic acid (Fig. S4), suggesting that human DDHD1 is phosphorylated at multiple minor sites when protein phosphatase is inhibited. The phosphorylation of minor sites may be highly sensitive to dephosphorylation by phosphatase.

### Evaluation of the effects of phosphorylation on PLA1 activity by phosphatase treatment and glutamic-acid-substituted DDHD1 mutants

The PLA1 activity of DDHD1 was measured by a method employing a fluorescent substrate (PED-A1). PED-A1 is a probe specific to PLA1 or lipase activities because the BODIPY FL acyl chain esterifies the *sn*-1 position and the short-chain alkyl moiety is ether-linked to the *sn*-2 position, which is resistant (noncleavable) to phospholipase A2. We confirmed this assay to be accurate and reliable to distinguish the small difference in activity because it can quantitatively measure linear changes in 50 ng of DDHD1 for 10 min (Fig. S5, *A* and *B*).

**Figure 3:**
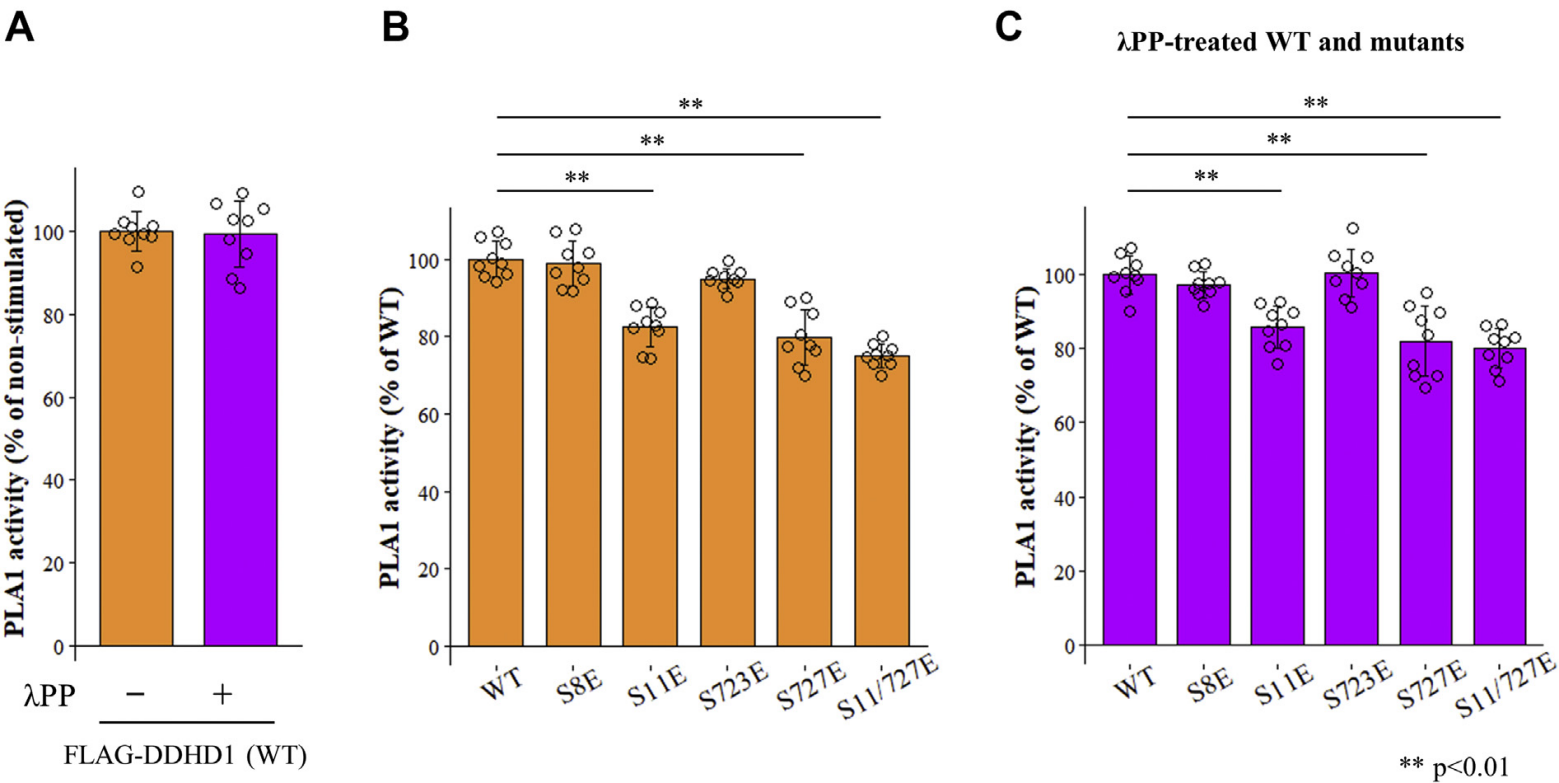
**Effects of phosphorylation on the PLA1 activity of DDHD1.***A*, purified recombinant FLAG-DDHD1 (WT) was treated with or without λPP and PLA1 activity was measured using a fluorescent substrate (PED-A1). *B* and *C*, the PLA1 activities of WT, S8E, S11E, S723E, S727E, and S11/727E were measured after treatment without (*B*) or with λPP (*C*). Fifty nanograms of purified WT and mutants quantified by bicinchoninic acid assay were used as enzymes for these assays. All three experiments were normalized by the averaged value of WT (*left*) in combination. Results are expressed as the mean ± SD of three experiments performed in triplicate (n = 9). Each individual point is also represented as a *small circle*. Statistical analyses were performed by the Student’s *t* test (*two asterisks*, *p* < 0.01).

To assess the effects of phosphorylation on PLA1 activity, purified FLAG-DDHD1 was treated with λPP. λPP did not markedly affect the PLA1 activity of WT (Fig. 3*A*), which may have been due to the offset caused by the multisite phosphorylation of DDHD1, which means that DDHD1 includes a mix of many major or minor phosphorylation sites up- or downregulating the PLA1 activity of DDHD1. Accordingly, the effects of the phosphorylation of individual sites need to be investigated.

As assessing the effects of the phosphorylation of individual sites in multisite phosphorylated proteins is difficult, we created several glutamic acid mutants (Fig. S1). The substitution of the serine residue to glutamic acid was postulated to mimic a constitutively phosphorylated state because of the addition of negative charges (phosphorylation-mimic mutants). These mutant enzymes were purified by immunoprecipitation with an anti-FLAG antibody, and PLA1 activity was measured by the method employing the fluorescent substrate.

The substitutions of Ser11, Ser727, and both by glutamic acid (S11E, S727E, and S11/727E) reduced PLA1 activity (Fig. 3*B*). In contrast, PLA1 activity was not affected by the substitution of Ser8 or Ser723 to glutamic acid (S8E and S723E, Fig. 3*B*). As such, the phosphorylation of Ser11 and S727, but not Ser8 or Ser723, reduced the PLA1 activity of DDHD1. However, the impact on PLA1 activity by phosphorylation was less than 25%.

The PLA1 activity of the phosphorylation-resistant form of DDHD1 (substitutions to alanine, S11A, S727A, and S11/727A) was not markedly different from that of WT (Fig. S4). These results indicated that Ser11 and Ser727 themselves are not essential for PLA1 activity, whereas the addition of negative charges by the phosphorylation of DDHD1 at Ser11 and Ser727 suppressed PLA1 activity.

To investigate the relationship between the phosphorylation mimics at Ser11/Ser727 and the phosphorylation of other sites, PLA1 activity was measured after WT and the phosphorylation mimic mutants of DDHD1 were treated with λPP (Fig. 3*C*). Compared with the enzymes not treated with λPP (Fig. 3*B*), the effects of substitution to glutamic acid were slightly attenuated by λPP. Therefore, the phosphorylation of Ser11 and Ser727 may negatively control the PLA1 activity of DDHD1 in coordination with the phosphorylation state at other sites influenced by them.

Taken together, the impact of phosphorylation on PLA1 activity was suggested to be less than 20%.

### Accumulation of phosphorylated DDHD1 in focal adhesions

**Figure 4:**
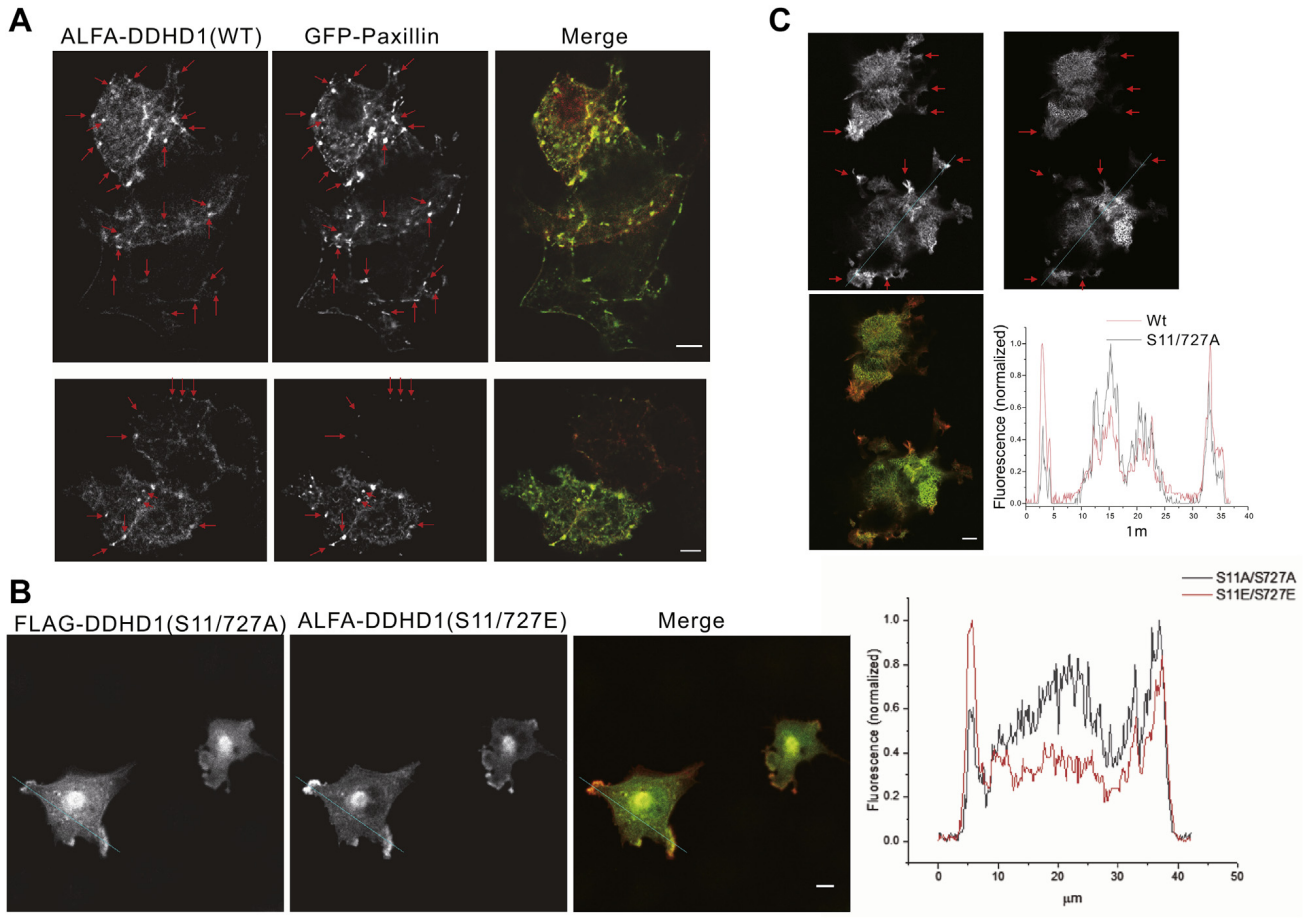
**Localization of phosphorylated DDHD at focal adhesions.***A*, ALFA-DDHD1 (WT) and GFP-Paxillin were expressed in COS-7 cells on retronectin-coated coverglasses and the cells were immunostained with tdTomato-anti-ALFA tag nanobody. The fluorescent signals of ALFA tag (*left*, *red*) and GFP (*right*, *green*) were acquired. Images 1.5 to 2.5 μm away from the glass surface are shown. Typical focal contacts are indicated by *arrows*. Bar, 5 μm. *B*, FLAG-DDHD1 (S11/727A) and ALFA-DDHD1 (S11/727E) were coexpressed in COS-7 cells and stained with FLAG-tag antibody (*red*) and anti-ALFA nanobody labeled with cfSGFP2 (*green*). The phospho-mimic form (S11/727E) was enriched at regions of focal adhesion, which was not observed with the phosphorylation-resistant mutant (S11/727A). The intensity map at the indicated line (*blue*) is also shown. *C*, immunostained FLAG-DDHD1 (WT) and ALFA-DDHD1 S11/S727A. Focal contacts are marked with *arrows* and the signal at the line (*blue*) was quantified. Bar, 5 μm.

Next, the effects of phosphorylation on the subcellular localization of DDHD1 were examined. When the cellular localization of DDHD1 was examined in COS-7 cells that were coated with a fibronectin fragment, retronectin, we noticed that the DDHD1 (WT) was located at the cellular contact sites attaching to the glass. Coexpression of DDHD1 with a focal adhesion marker, paxillin α (30), revealed that the spots were indeed focal adhesions (Fig. 4*A*). We then confirmed if this localization pattern was associated with the phosphorylation status of DDHD1. Of note, the apparent concentration of WT at focal adhesions was also observed with the phospho-mimic form S11/727E (*right*, Fig. 4*B* and intensity map), but not with the phosphorylation-resistant mutant S11/S727A (*left*). A similar difference was noted when the alanine mutant expression profile was compared with that of WT (Fig. 4*C*). These findings are consistent with our observation that Ser11 and Ser727 of DDHD1 are nearly always phosphorylated (Fig. 1*B*). These results suggested that the phosphorylation at Ser11 and Ser727 regulates the targeting of DDHD1 to focal contacts.

### Identification of responsible kinases of DDHD1

To identify the protein kinases that phosphorylate DDHD1, we predicted candidates from amino acid sequences around identified phosphorylation sites using site-specific kinase-substrate prediction algorithms such as NetPhos 3.1 Server, Scansite 4.0 (https://scansite4.mit.edu/4.0/#home), and iGPS (http://igps.biocuckoo.org/). These software programs predicted several protein kinases, including cyclin-dependent kinases (CDKs) and glycogen synthase kinase-3 (GSK-3), with relatively high scores (NetPhos 3.1, Table S1).

**Figure 5:**
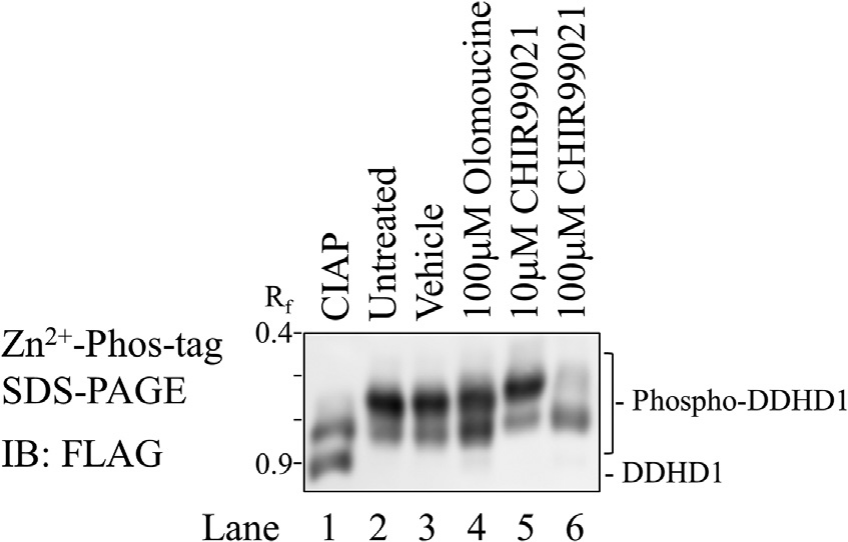
**Effects of protein kinase inhibitors on the phosphorylation of DDHD1.** FLAG-DDHD1-expressing PANC1 cells were cultured in the absence (lanes 1–3) or presence of 100 μM olomoucine (lane 4) or 10 (lane 5), or 100 μM CHIR99021 (lane 6) for 2 days with growth medium containing 10% FBS. Cell extracts from vehicle-treated cells were incubated with (lane 1) or without calf intestine alkaline phosphatase (CIAP) (lane 2). Cell extracts were analyzed by Zn^2+^ Phos-tag SDS-PAGE following immunoblot analysis (IB). The R_f_ value of 1.0 is defined as the position of bromphenol blue dye. Results are from one experiment representative of three independent experiments.

The effects of the specific inhibitor for CDKs and GSK-3 were investigated. The treatment of PANC1 cells expressing FLAG-DDHD1 with olomoucine (CDK inhibitor) resulted in faster migrating bands corresponding to partially unphosphorylated forms (Fig. 5, lane 4). When the same cells were cultured in the presence of CHIR99021 (GSK-3 inhibitor), the ratio of fast/slow migrating bands markedly increased in a dose-dependent manner (Fig. 5, lanes 5 and 6). This suggested that inhibitors of CDKs and GSK-3 reduced the phosphorylation of human DDHD1.

As relatively high doses of inhibitors of protein kinases were required to reduce the phosphorylation of DDHD1, the specificity of the inhibitors was of concern. Therefore, we investigated the effects of knockdown of GSK3β or CDK1 using miRNA expression vectors. miRNA for GSK3β or CDK1 reduced the expression of the protein kinases; however, the knockdown did not affect the phosphorylation of DDHD1 in HEK293 cells (Fig. S6). This suggested that multiple kinases targeted the same phosphorylation site of DDHD1 in intact cells. This was consistent with the NetPhos prediction and requirement of high doses of the inhibitors.

**Figure 6:**
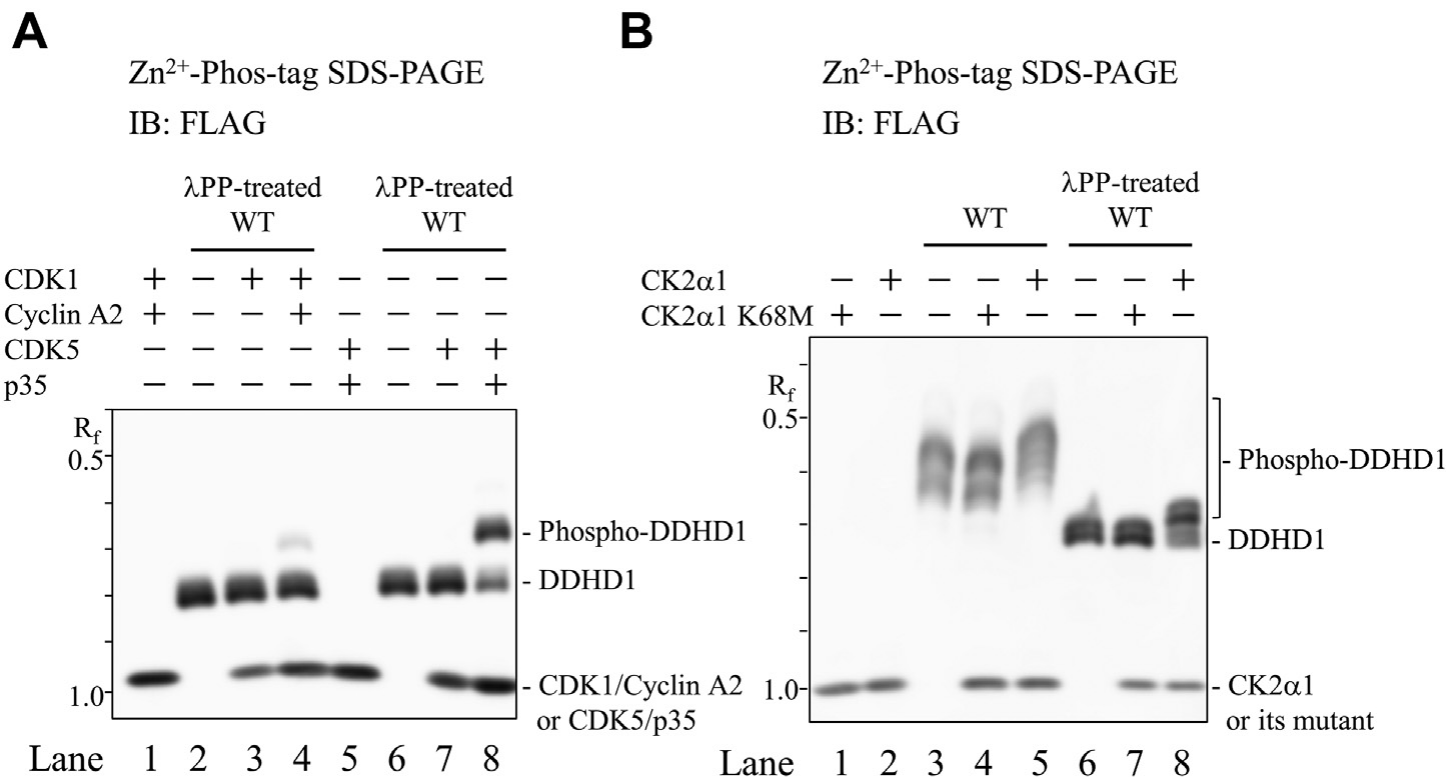
**Phosphorylation of DDHD1 by CDK1, CDK5, and CK2α1.***A*, Phosphorylation by CDK1 or CDK5. The dephosphorylated form of purified recombinant FLAG-DDHD1 (λPP-treated WT, lanes 2–4 and 6–8) or TBS (none, lanes 1 and 5) was incubated in combination with purified FLAG-tagged CDK1 or CDK5, and their activators, cyclin A2 or p35, at 25 °C for 180 min. *B*, Phosphorylation by CK2α1, recombinant DDHD1 (WT, lanes 3–5), its dephosphorylated form (λPP-treated WT, lanes 6–8), or TBS (none, lanes 1 and 2) was incubated in combination with purified FLAG-tagged CK2α1 or its kinase-inactive form K68M at 25 °C for 180 min. The incubated mixtures containing equivalent amounts of DDHD1 were analyzed by Zn^2+^ Phos-tag SDS-PAGE following immunoblot analysis using anti-FLAG M2 antibody. The R_f_ value of 1.0 is defined as the position of bromphenol blue dye. Results are from one experiment representative of three independent experiments.

To obtain clearer evidence, we conducted an *in vitro* kinase assay employing purified recombinant protein kinases. We selected CDKs and GSK3 because they were predicted to be involved kinases (Table S1). After the substrate DDHD1 was dephosphorylated by pretreatment with λPP, DDHD1 was incubated with purified protein kinases and ATP, and the reaction products were analyzed by Phos-tag SDS-PAGE (Fig. 6*A*). When CDK1 was used as the candidate kinase, up-shifted bands of DDHD1 were observed in the presence of cyclin A2 (Fig. 6*A*, *left*); however, a new and slow-migrating band was very faint (Fig. 6*A*, lane 4). In contrast, a very strong band shift was observed when cyclin-dependent kinase 5 (CDK5) was used as the candidate kinase in the presence of p35 (Fig. 6*A*, lane 8). In both cases, these up-shifted bands were not observed in the absence of cyclin A2 and p35, respectively (Fig. 6*A*, lanes 3 and 7). Cyclin A2 and p35 themselves have no kinase activity. Therefore, DDHD1 was phosphorylated by activated CDK1 and CDK5 because cyclin A2 and p35 are activators of CDK1 and CDK5, respectively. In addition, CDK5/p35 more strongly phosphorylated DDHD1 than CDK1/cyclin A2 with the same amount of kinase/activator (Fig. 6*A*, lanes 4 and 8).

Changes in the migration of DDHD1 were not observed when DDHD1 was used without pretreatment with λPP as a substrate. The underlying reason may be that Ser8, Ser11, and Ser727 of DDHD1 were already phosphorylated in HEK293 cells (Fig. 1). CDK1/cyclin A2 and CDK5/p35 may be involved in the phosphorylation of these sites in DDHD1 under the growth conditions of HEK293 cells.

A similar analysis was conducted using casein kinase 2, alpha 1 (CK2α1) (Fig. 6*B*) because CK was another predicted candidate. When λPP-untreated DDHD1 was used as a substrate, up-shifted bands were observed by CK2α1 (Fig. 6*B*, lane 5), but not by the kinase-inactive isoform CK2α1 K68M (lane 4) in Phos-tag SDS-PAGE. Thus, site(s) phosphorylated by CK2α1 may differ from those in DDHD1 by other kinases, including CDK1/cyclin A2 and CDK5/p35, under the growth conditions of HEK293 cells.

The pretreatment with λPP increased the mobility of DDHD1 (Fig. 6*B*, lanes 3 and 6), whereas that with CK2α1 induced an up-shift of the bands of DDHD1 (Fig. 6*B*, lane 8); however, kinase-inactive isoform CK2α1 K68M did not (lane 7). Furthermore, CK2α1 phosphorylated DDHD1, but treatment with CK2α1 did not reverse the phosphorylation state before the pretreatment with λPP, again suggesting that the site(s) phosphorylated by CK2α1 differ from those in DDHD1 by kinases under the growth conditions of HEK293 cells.

However, the treatment of the dephosphorylated form of DDHD1 with purified GSK3α or GSK3β did not induce up-shifted bands (Fig. S7, *A* and *C*), although a high dose of the GSK-3 inhibitor CHIR99021 resulted in faster migrating bands, as described before. As such, GSK may phosphorylate DDHD1 *in vivo*, but not *in vitro*, which may have been due to priming phosphorylation being necessary for the phosphorylation of GSK-3β (31). Recombinant ERK2 and p38 also did not induce up-shifted bands (Fig. S7, *B* and *C*). These results suggest that ERK2 and p38 need to be phosphorylated by upstream protein kinases (32). Collectively, the present results suggest that CDK1/cyclin A2, CDK5/p35, and CK2α1 are the kinases responsible for the phosphorylation of human DDHD1.

### Identification of phosphorylation sites of DDHD1 targeted by responsible kinases

**Figure 7:**
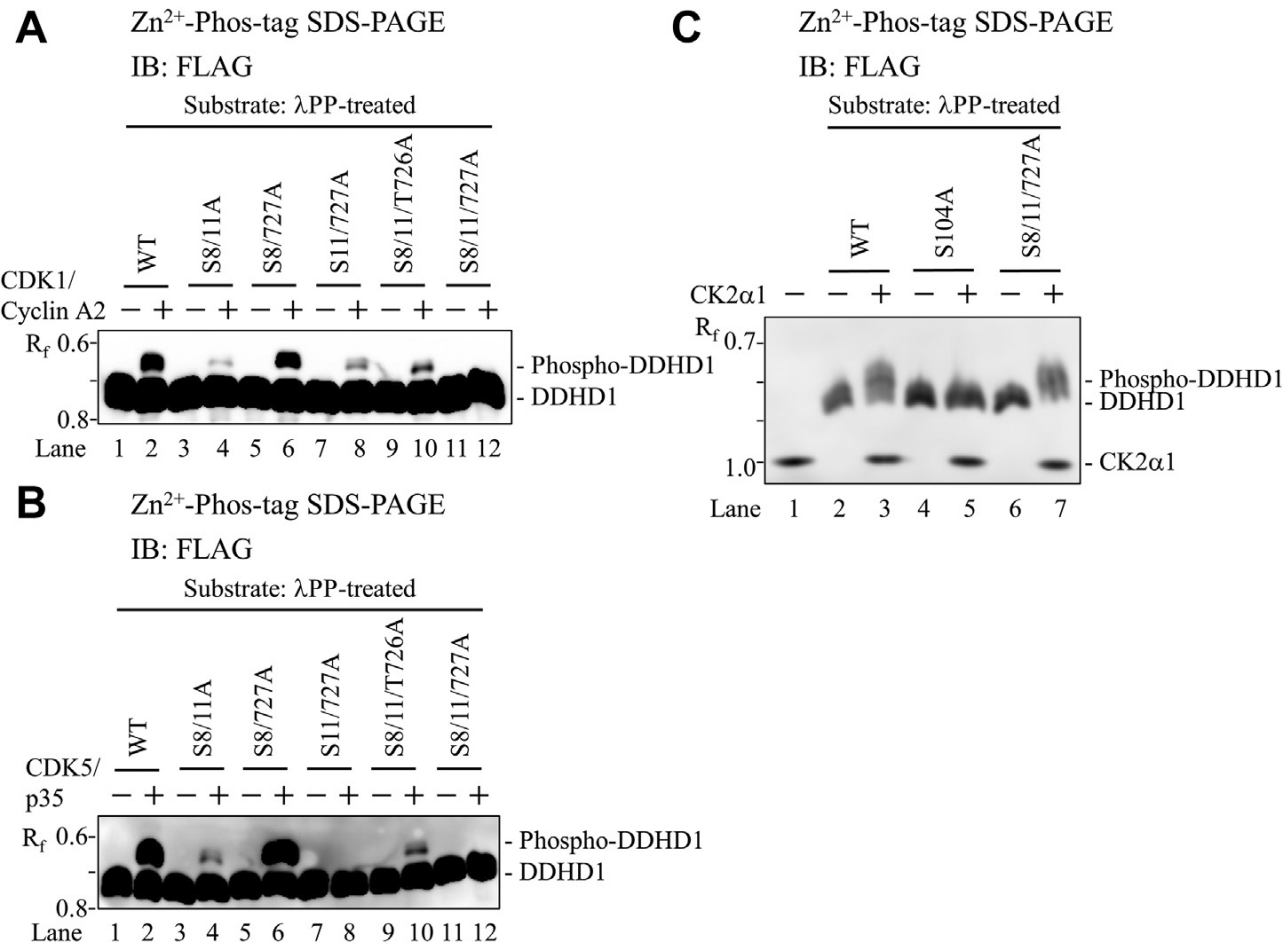
**Identification of phosphorylation sites of DDHD1 targeted by CDK1, CDK5, and CK2α1.***A* and *B*, Phosphorylation by CDK1 or CDK5. The dephosphorylated form of purified recombinant FLAG-DDHD1 (WT) or its alanine mutants (S8/11A, S8/727A, S11/727A, S8/11/T726A, and S8/11/727A) was incubated with or without purified FLAG-tagged CDK1/cyclin A2 (*A*) or CDK5/p35 (*B*) at 25 °C for 150 min. *C,* Phosphorylation by CK2α1, the dephosphorylated form of purified recombinant FLAG-DDHD1 (WT) or its alanine mutants (S104A and S8/11/727A) was incubated with or without purified FLAG-tagged CK2α1 at 25 °C for 180 min. The incubated mixtures containing equivalent amounts of DDHD1 were analyzed by Zn^2+^ Phos-tag SDS-PAGE following immunoblot analysis using anti-FLAG M2 antibody. Blots were exposed to ECL reagent in order to detect up-shifted bands by CDK1/cyclin A2 or CDK5/p35. The R_f_ value of 1.0 is defined as the position of bromphenol blue dye. Results are from one experiment representative of three independent experiments.

We next investigated the phosphorylation site(s) of DDHD1 by each protein kinase. We performed an *in vitro* kinase assay followed by Phos-tag SDS-PAGE analysis employing DDHD1 mutants with multiple mutated phosphorylation sites and purified protein kinases. All experiments were performed using the dephosphorylated forms of WT and mutant proteins as a substrate by pretreatment with λPP. The incubation of WT DDHD1 with CDK1/cyclin A2 and ATP resulted in an up-shifted band (Fig. 7*A*, lane 2). However, when S8/11/727A was used as a substrate, the bands up-shifted by CDK1/cyclin A2 disappeared (Fig. 7*A*, lane 12), suggesting that CDK1/cyclin A2 phosphorylated DDHD1 at Ser8, Ser11, or Ser727. When the S8/11A, S8/727A, or S11/727A mutant was used, up-shifted bands were observed (Fig. 7*A*, lanes 4, 6, and 8). As Ser727, Ser11, and Ser8 are intact in the S8/11A, S8/727A, and S11/727A mutants, respectively, CDK1/cyclin A2 phosphorylated the intact serine. However, the degrees of phosphorylation by CDK1/cyclin A2 differed at each serine. The intensity of up-shifted bands was high with the S8/727A mutant (Fig. 7*A*, lane 6), whereas only small bands were detected with the S8/11A and S11/727A mutants (Fig. 7*A*, lanes 4 and 8). These results strongly suggest that CDK1/cyclin A2 prefers to phosphorylate Ser11 over Ser8 and Ser727 in DDHD1.

Similar analyses employing CDK5/p35 and each DDHD1 mutant were conducted (Fig. 7*B*). When WT DDHD1 was used, up-shifted bands were observed in the presence of CDK5/p35 and ATP (Fig. 7*B*, lane 2). However, when S11/727A and S8/11/727A were used, the up-shifted bands disappeared (Fig. 7*B*, lanes 8 and 12), suggesting that CDK5/p35 phosphorylated DDHD1 at Ser11 and Ser727. Based on the intensity of the up-shifted bands for each mutant (Fig. 7*B*, lanes 4 and 6), CDK5/p35 prefers to phosphorylate Ser11 over Ser727 in DDHD1. These results reflect differences in the specificity of the phosphorylation site by each CDK isoform. However, as Ser11 and Ser727 were both preferred phosphorylation sites over Ser8 because the fast migrating band increased with the S11/727A mutant (Fig. 1*B*, lane 10) compared with that of S727A and S8/727A mutants (Fig. 1*B*, lanes 7 and 11), the physiology of DDHD1 affected by CDK1 or CDK5 in HEK293 cells may mainly be due to a difference in the strength of kinase activity.

When the behaviors of S8/11/T726A and S8/11/727A were compared (Fig. 7, *A* and *B*, lanes 9–12), CDK1/cyclin A2 and CDK5/p35 both phosphorylated Ser727, but not Thr726, consistent with the results shown in Figure 1*B*.

Treatment of the dephosphorylated form of WT with CK2α1 and ATP resulted in an up-shifted band (Fig. 7*C*, lanes 2 and 3). Similar results were observed when the S8/11/727A mutant was employed as a substrate (Fig. 7*C*, lanes 6 and 7), suggesting that CK2α1 phosphorylated DDHD1 at serine/threonine residue(s) other than Ser8, Ser11, and Ser727. Therefore, we considered potential phosphorylation site(s) based on the MALDI-TOF MS/MS analysis (Table 1). Although other mutants exhibited no difference from WT like S8/11/727A, the use of the S104A mutant as a substrate resulted in the disappearance of its up-shifted band (Fig. 7*C*, lanes 4 and 5), suggesting that CK2α1 phosphorylated the Ser104 residue of DDHD1. However, the Mascot ion score of peptides containing single-phosphorylated Ser104 was not significant. Therefore, Ser104 is a novel, but minor phosphorylation site in HEK293 cells. This was consistent with the migration of the S104A mutant in Phos-tag SDS-PAGE not being markedly different from that of WT under the growth conditions of HEK293 cells.

### Effects of CDK5/p35 on the PLA1 activity of DDHD1

**Figure 8:**
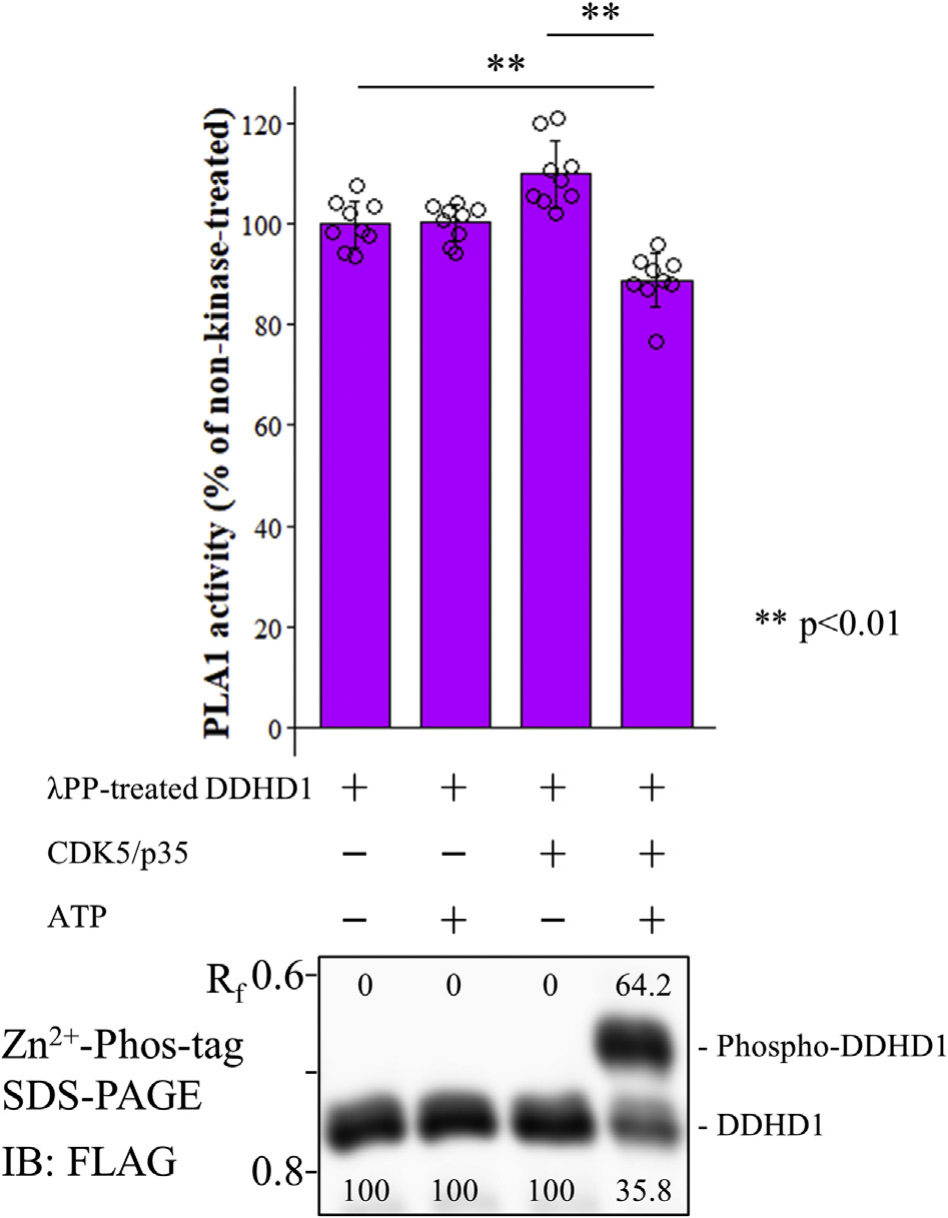
**Effects of CDK5/p35 on the PLA1 activity of DDHD1.** The dephosphorylated form of purified recombinant FLAG-DDHD1 was incubated with or without purified FLAG-tagged CDK5/p35 or ATP at 25 °C for 360 min, and the PLA1 activity of reaction mixtures containing 50 ng of DDHD1 was compared. All three experiments normalized by the averaged value of WT without CDK5/p35 and ATP (*left-most*) are combined. Results are expressed as the mean ± SD of three experiments performed in triplicate (n = 9). Each individual point is also represented as a *small circle*. Statistical analyses were performed by the Student’s *t* test (*two asterisks*, *p* < 0.01). The *lower panel* shows phosphorylation states at the time of the PLA1 activity measurement detected by Zn^2+^ Phos-tag SDS-PAGE followed by immunoblot analysis (IB) using anti-FLAG M2 antibody, and the *numeric characters* below each band indicate the abundance ratio of DDHD1 and phosphorylated DDHD1. The R_f_ value of 1.0 is defined as the position of bromphenol blue dye.

We investigated whether CDK5/p35 regulates the PLA1 activity of DDHD1 by phosphorylation because glutamic acid mutants of Ser11 and Ser727 targeted by CDK5/p35 reduced the PLA1 activity (Fig. 3). We measured the PLA1 activity of DDHD1 with or without treatment with a combination of CDK5/p35 and ATP *in vitro*. The PLA1 activity of DDHD1 was significantly reduced by the treatment with CDK5/p35 and ATP (Fig. 8, upper panel). However, individual treatment with CDK5/p35 or ATP did not affect PLA1 activity. The phosphorylation status of DDHD1 under these conditions was assessed; only the treatment with CDK5/p35 and ATP induced phosphorylation (Fig. 8, lower panel). These results demonstrated that the inhibition of PLA1 activity was due to the phosphorylation of DDHD1, but not the binding of CDK5/p35 or ATP to DDHD1. As phosphorylation was not complete (64.2%) and CDK5/p35 phosphorylated Ser11 and Ser727 (Fig. 8), the degree of inhibition (11.2%) may be consistent with the activity of the S11/727E mutant (reduced by approximately 20%, Fig. 3). This again suggested that the impact of phosphorylation on the PLA1 activity is less than 20%.

**Figure 9:**
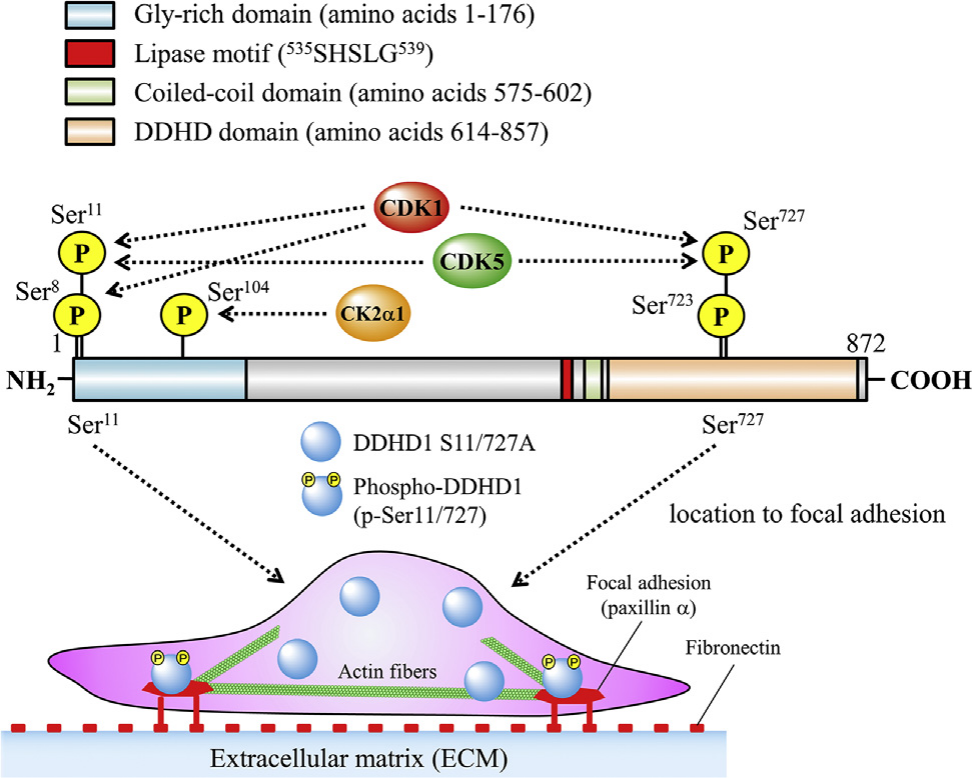
**Summary of the phosphorylation of human DDHD1, potential responsible protein kinases, and possible physiological function.** The lipase consensus sequence and DDHD1 domain are shown in *red* and *light orange*, respectively. The glycine-rich and coiled-coil domains are shown in *pale blue* and *green*, respectively. Numbers, such as 1 and 872, are those of amino acid residues. The phosphorylation sites identified in the present study are depicted as P in *yellow circles*. The phosphorylation sites by CDK1/cyclin A2, CDK5/p35, and CK2α1 are indicated. Translocation of DDHD1 to focal adhesions by phosphorylation at Ser11 and Ser727 is depicted.

## Discussion

Protein phosphorylation is the most common posttranslational modification of protein(s) and functions as a molecular switch for their regulation (28). This modification is reversibly regulated by protein kinase(s) and phosphatase(s). In the present study, we demonstrated that human DDHD1 is a multisite phosphorylated protein. Mass spectrometry and Phos-tag SDS-PAGE with alanine-substituted mutants revealed that DDHD1 has four major phosphorylation sites: Ser8, Ser11, Ser723, and Ser727 (Fig. 9). Based on *in vitro* kinase assays and Phos-tag SDS-PAGE, Ser8, Ser11, and Ser727 are phosphorylated by the CDK1/cyclin A2 complex (Fig. 6). In addition, Ser11 and Ser727 are phosphorylated by the CDK5/p35 complex (Fig. 6). CDK1 and CDK5 both have phosphorylation preferences for Ser11 in DDHD1. Another kinase, CK2α1, phosphorylated Ser104 (Fig. 6); however, phosphorylation at this site was minor in HEK293 cells cultivated in growth medium. GSK-3 may also be involved in the phosphorylation of DDHD1 because unphosphorylated forms were increased by treatment with the GSK-3 inhibitor CHIR99021. Hyperphosphorylation of DDHD1 was observed when DDHD1-expressing cells were treated with a protein phosphatase inhibitor. Collectively, these results suggest reversible regulation through the phosphorylation–dephosphorylation cycle of DDHD1 by multiple protein kinases and phosphatases.

The serine and threonine residues of DDHD1 are conserved among several mammals, including humans, cows, and rodents; however, the phosphorylation of these amino acid residues may differ. A previous study revealed that bovine DDHD1 is phosphorylated by CK2 at Ser93, Ser105, and Ser716 *in vitro* (33), which correspond to Ser92, Ser104, and Ser713, respectively, in human DDHD1. Human DDHD1 was only phosphorylated at Ser104, not at Ser92 or Ser713, in growing cells cultured with FBS (Table 1). In addition, the *in vitro* kinase assay revealed that CK2α1 phosphorylated Ser104 (Fig. 7*C*). Even Ser104 may be a minor phosphorylation site because Phos-tag SDS-PAGE of S104A did not markedly alter mobility. On the other hand, Ser730 in the bovine enzyme was phosphorylated by ERK2, whereas the corresponding Ser727 in the human enzyme was phosphorylated by CDKs (Fig. 7, *A* and *B*) and not ERK2 (Fig. S7). The present study also demonstrated that Ser8, Ser11, and Ser723 in human DDHD1 are major phosphorylation sites; however, the phosphorylation of the corresponding sites in the bovine enzyme was not identified in the previous study (33). Therefore, although Ser104 (same responsible kinase, CK2) and Ser727 (different kinases, human CDKs; bovine ERK2) are conserved as phosphorylation sites between human and bovine DDHD1, Ser8, Ser11, Ser723 (the former, human), Ser92, and Ser713 (the latter, bovine) may be animal-specific phosphorylation sites. The mouse DDHD1 protein also lacks the region containing Ser8 and Ser11. The phosphorylation sites and responsible kinases in DDHD1 are considered to be highly diverse among mammals.

The phosphorylation sites of DDHD1 may also be different for each cell type even in humans. All phosphopeptides except for those containing Ser11, Ser8/11, and Ser332 (not Ser8) were identified in both HEK293 and PANC1 cells (Table 1). Although Ser11 was not identified as a phosphorylation site of PANC1 cells in this experiment, Ser11 is also a phosphorylation site in PANC1 cells, as shown in Fig. S3. Ser332 may be a PANC1 cell-specific phosphorylation site because it was repeatedly identified only in PANC1 cells and not in HEK293.

As the inhibitor of GSK-3β (CHIR99021) up-shifted bands in Phos-tag SDS-PAGE, we considered GSK-3β to also be a responsible kinase for human DDHD1 (Fig. 5). The consensus sequence for phosphorylation by GSK-3β is S/T-X-X-X-S/T-(P) and the phosphorylation of Ser723 was detected; therefore, the region ^723^SPVTSP^728^ may be one of the phosphorylation sites. However, we were unable to confirm the phosphorylation of human DDHD1 in an *in vitro* kinase assay employing recombinant GSK-3β (Fig. S7). Primed phosphorylation of Ser727 may be needed for the phosphorylation of Ser723 by GSK-3β because primed phosphorylation at the Ser/Thr residue located 4 amino acids downstream by another kinase(s) was previously reported to accelerate the rate of phosphorylation by GSK-3β (31). Kinases beyond those identified in the present study may also phosphorylate DDHD1 in intact cells because DDHD1 is a highly phosphorylated protein (Table 1, and Figs. S4 and S6).

We examined the effects of phosphorylation(s) on PLA1 activity. Precisely evaluating phosphorylation is difficult because human DDHD1 is phosphorylated at multiple sites, even in cells cultivated in growth medium, and multisite phosphorylation(s) may exert offset effects on PLA1 activity. The nonselective dephosphorylation of purified DDHD1 by λPP did not markedly alter the PLA1 activity (Fig. 3*A*). We employed phosphorylation mimic mutants in which an individual serine was substituted to glutamic acid because this mutation gives a negative charge and is hypothesized to mimic a constitutively phosphorylated state. The PLA1 activity of S11E, S727E, and S11/727E was lower than that of WT DDHD1 (Fig. 3*B*), suggesting that the phosphorylation(s) of Ser11 and S727 reduced this activity. Reduced activity was significant, but the potency was not large and the degree was less than 20%.

We next assessed the relationship between the phosphorylation(s) of Ser11/Ser727 and PLA1 activity using the *in vitro* kinase reaction by CDK5/p35. Approximately 64.2% of DDHD1 was phosphorylated by the kinase reaction, and the activity of the 100% phosphorylated form was calculated to be 70.1% of the dephosphorylated form (third bar graph) in comparison with the activity of CDK5/p35-treated DDHD1 without ATP as a control (Fig. 8). The extent of the reduction in activity by Ser11/Ser727 phosphorylation may correlate with that by the substitution of glutamate in Ser11/Ser727 (Fig. 3). This suggested that the effects of glutamate need to be equivalent to those of phosphoserine and that the impact of phosphorylation on PLA1 activity is not high (~30% reduction). In the case of bovine DDHD1, phosphorylation by ERK2 did not affect PLA1 activity, whereas that by CK2 induced an approximately 50% reduction (33).

As phosphorylation(s) did not markedly affect PLA1 activity, phosphorylation(s) may affect other properties of DDHD1. We thus examined the effects of phosphorylation on its subcellular localization. Phosphorylation at Ser11 and Ser727 was strongly suggested to facilitate localization of the enzyme to focal adhesions (Figs. 4 and 9). Focal adhesions are subcellular structures that mediate signaling in response to adhesion of the extracellular matrix (ECM) (34). Focal adhesions serve as the mechanical linkages to the ECM and as a biochemical signaling hub to concentrate and direct numerous signaling proteins at sites of integrin binding and clustering. Focal adhesion complexes contain various signaling proteins that play an essential role in directing cell motility, growth, differentiation, survival, and invasion. The physiological role of the location of phosphorylated DDHD1 has not been elucidated because this is the first report for DDHD1. However, as both potential substrates (PA and phosphatidylinositol (and phosphoinositides)) and products (lysophosphatidic acid (LPA) and LPI) are involved in cell growth and survival (4, 5, 12), DDHD1 may regulate signaling in focal adhesions. In addition, mutation(s) in human DDHD1 cause the neurodegenerative disorder HSP (*SPG28* subtype) (16, 17, 18, 19, 20), thus neuronal death may be involved. This study presented an important question to be solved in the future.

## Experimental procedures

### Materials

Oligonucleotides for the expression of the epitope-tagged DDHD1 mutant in HEK293 and PNAC1 cells described below were synthesized by Invitrogen. Sequencing-grade modified trypsin and AspN were purchased from Promega. High-purity MS grade 2, 5-dihydroxybenzoic acid (DHB) was from Shimadzu. α-Cyano-4-hydroxycinnamic acid (α-CHCA) was from Sigma. Phos-tag acrylamide was from Wako. The mixture of protease inhibitors containing 4-(2-aminoethyl) benzenesulfonyl fluoride, aprotinin, bestatin, E-64, leupeptin, and pepstatin A was from Calbiochem. Olomoucine was from Sigma. CHIR99021 was from Wako. Anti-mouse IgG antibodies conjugated with horseradish peroxidase were from Dako. All other reagents were from Wako, unless otherwise mentioned. All other compounds were of reagent grade or higher. The expression plasmid for paxillin α (35) was a generous gift of Dr M. Nishita (Fukushima Medical University).

### Cell culture

HEK293, COS7, and PANC1 cells were maintained in high-glucose Dulbecco’s modified Eagle’s medium (D-MEM, Wako) containing 10% fetal calf serum and 1% penicillin/streptomycin (Gibco). Retroviral package GP2-293 cells, which stably express the *gag* and *pol* genes of vesicular stomatitis virus, were maintained in D-MEM containing 10% fetal calf serum, 1% penicillin/streptomycin, 4 mM L-glutamine, and 1 mM sodium pyruvate. Cells were grown on a monolayer at 37 °C in a humidified atmosphere with 5% CO_2_.

### Plasmid preparation

The cDNA encoding full-length human DDHD1 with N-terminal FLAG or ALFA (36)-tag was subcloned into the pcDNA4/TO expression vector (Invitrogen) or pQCXIP retroviral vector (Clontech), as in our previous study (12). In the *in vitro* kinase assay, the cDNA encoding human CDK1, cyclin A2, CDK5, p35, or CK2α1 with a FLAG-tag was amplified by PCR using cDNA derived from HeLa cells as a template (Fig. S1). All were inserted into the pcDNA4/TO expression vector (Invitrogen). All constructs were verified by DNA sequencing.

### Site-directed mutagenesis of human DDHD1 and CK2α1

The human DDHD1 mutants of S8A, S11A, S104A, S723A, T726A, S727A, S8E, S11E, S723E, and S727E, and the human CK2α1 K68M kinase-dead mutant were generated using the QuikChange II site-directed mutagenesis kit (Stratagene) according to the manufacturer’s instructions using pcDNA4/TO/FLAG-DDHD1 and pcDNA4/TO/FLAG-CK2α1 plasmids as templates, respectively. The primer sets utilized are described in Table 2. All constructs were verified by sequencing analysis.

**Table 2:** Primer sets used for site-directed mutagenesis

Primer set	Sense primer sequence (5' to 3')	Antisense primer sequence (5' to 3')
DDHD1 mutants		
S8A	GGGCCGCGGGGCCCCACGGAG	CTCCGTGGGGCCCCGCGGCCC
S11A	CGGGTCCCCACGGGCCCCCGAGCATAAC	GTTATGCTCGGGGGCCCGTGGGGACCCG
S104A	CGCTGCGCTACTACGCCGAGGGTGAGAGCG	CGCTCTCACCCTCGGCGTAGTAGCGCAGCG
S723A	GAATGAAGGCATTTCAACCATACCAGCCCCTGTGACCTCA	TGAGGTCACAGGGGCTGGTATGGTTGAAATGCCTTCATTC
T726A	CATACCAAGCCCTGTGGCCTCACCAGTTTTGTC	GACAAAACTGGTGAGGCCACAGGGCTTGGTATG
S727A	CCAAGCCCTGTGACCGCACCAGTTTTGTCCC	GGGACAAAACTGGTGCGGTCACAGGGCTTGG
S8E	CCGGGCCGCGGGGAGCCACGGAGCCCC	GGGGCTCCGTGGCTCCCCGCGGCCCGG
S11E	GCGGGTCCCCACGGGAGCCCGAGCATAACGG	CCGTTATGCTCGGGCTCCCGTGGGGACCCGC
S723E	GAGAATGAAGGCATTTCAACCATACCAGAGCCTGTGACCTCACCA	TGGTGAGGTCACAGGCTCTGGTATGGTTGAAATGCCTTCATTCTC
S727E	TACCAAGCCCTGTGACCGAGCCAGTTTTGTCCCGCCG	CGGCGGGACAAAACTGGCTCGGTCACAGGGCTTGGTA
CK2α1 mutant		
K68M	GCCATCAACATCACAAATAATGAAAAAGTTGTTGTTATGATTCTCAAGCCAGTAAAAAA	TTTTTTACTGGCTTGAGAATCATAACAACAACTTTTTCATTATTTGTGATGTTGATGGC

For all primers, mutagenized positions are underlined.

### Protein expression in mammalian cells

The pcDNA4/TO-based epitope-tagged human DDHD1, protein kinases, or their mutant expression vectors were transfected into HEK293 or PANC1 cells using Lipofectamine 2000 (Invitrogen) or Calfectin (SignaGen) according to the manufacturer’s protocol. The pQCXIP-based human FLAG-DDHD1 expression vector was cotransfected with pVSV-G (Clontech) into GP2-293 cells to produce the retrovirus. After being incubated in medium containing retroviral particles for 2 to 3 days, the target cells were treated with puromycin (Sigma) for 2 weeks to select cells stably expressing DDHD1 according to the manufacturer’s protocol. The stable DDHD1-expressing cell lines by retroviral vector transfection were mainly used in MALDI-TOF MS/MS and Phos-tag SDS-PAGE experiments with protein kinase inhibitors.

### Purification of recombinant proteins

1 × 10^7^ cells expressing FLAG-tagged proteins were harvested and lysed in ice-cold immunoprecipitation buffer (50 mM Tris-HCl pH 7.4, 150 mM NaCl, 1 mM EDTA, 1% (w/v) NP-40, and protease inhibitors) at 4 °C. The lysate was centrifuged for 30 min at 15,000 rpm. The resulting supernatant with anti-FLAG M2 affinity gel (Sigma) was incubated with gentle rocking at 4 °C overnight. The gel was washed three times with TBS buffer, pH 8.0, and the enzyme was eluted from the gel by competitive replacement with 100 μg/ml of FLAG peptide (Sigma) according to the manufacturer’s instructions. The amount of purified protein was measured using Bicinchoninic Acid Solution reagent (Pierce). Purified proteins were used to assess phospholipase enzymatic activity or in the *in vitro* kinase assay as described below.

### Dephosphorylation of the purified recombinant enzyme

Prior to examining the phosphorylation of recombinant DDHD1 or its mutants (in most experiments), we removed the esterified phosphate groups introduced into HEK293 cells (1 × 10^7^) by attaching the enzyme to anti-FLAG M2 affinity gels, washing them with TBS buffer, at pH 8.0 three times, and incubating them at 30 °C for 1 h with an excess amount of lambda protein phosphatase (λPP, New England Biolabs). After the incubation, enzyme-containing gels were washed three times with TBS buffer, at pH 8.0 to remove λPP, and the enzyme was eluted as described above.

### Identification of phosphorylation sites in human DDHD1 by MALDI-TOF MS/MS

After treating 6 × 10^7^ cells expressing FLAG-DDHD1 with or without 1 μM okadaic acid (Sigma) at 37 °C for 4 h, we used SDS-PAGE to purify the DDHD1 protein immunoprecipitated with anti-DYKDDDDK (FLAG) monoclonal antibody (clone 2H8, TransGenic) conjugated to Dynabeads protein G (Invitrogen) and then digested aliquots of the purified enzyme with trypsin or AspN using a procedure modified from a previously published protocol (37). Briefly, the gel band containing phosphorylated DDHD1 was excised, dehydrated for 10 min in acetonitrile (ACN), and dried in a VC-36R Spin Dryer Lite (TAITEC). Pieces of the dried gel were incubated at 4 °C for 30 min in 50 mM NH_4_HCO_3_ containing trypsin (10 μg/ml) or AspN (10 μg/ml). The temperature was increased to 37 °C and the incubation was continued overnight. After the incubation, digested DDHD1 was extracted from gel pieces three times using 0.1% trifluoroacetic acid (TFA) in 50% ACN. The extracted peptides were pooled, dried, and then dissolved in 0.1% TFA or 300 mg/ml of DHB in 80% ACN/0.1% TFA. Phosphopeptides in the former and latter solvents were further enriched with the Magnetic Phosphopeptide Enrichment Kit (Clontech) based on IMAC and Nutips (Glygen) consisting of TiO_2_, ZrO_2_, or a mixture of both, respectively, according to the manufacturer’s instructions, but with slight modification. The phosphopeptide solutions were dried, reconstituted in 0.1% TFA, and desalted using ZipTip C_18_ tips (Millipore). Purified peptides were released in 50% ACN/0.1% TFA, which was mixed with an equal amount of 10 mg/ml of α-CHCA or 20 mg/ml of DHB matrix solution in 50% ACN/0.1% TFA containing 1% phosphoric acid (Sigma) for analysis by MALDI-TOF MS on AXIMA Performance (Shimadzu). The instrument was calibrated using a standard of bradykinin fragment 1 to 7 ([M + H]^+^ 757.40, Sigma), N-acetyl-renin substrate ([M + H]^+^ 1800.94, Sigma), and ACTH fragment 7 to 38 ([M + H]^+^ 3657.93, Sigma) for MS and MS/MS. MS and MS/MS analyses were performed in the positive reflector mode using Shimadzu Biotech MALDI–MS Launchpad v. 2.8.4. Mono and average isotopic peak lists were generated by MALDI-MS software. MS data obtained from the purified phosphopeptides of DDHD1 were compared with a theoretical peptide mass fingerprint of DDHD1 with phosphorylated serine and threonine sites by MASCOT website (http://www.matrixscience.com/) or software (version 2.5, Matrix Science). Peptides matching the masses of predicted phosphopeptides were preferentially selected for fragmentation by a form of postsource decay without using collision gas. Initial MS/MS data were examined for the presence of a neutral loss of 98 Da corresponding to the phosphate moiety and selected phosphopeptides were directed to collect more thorough data to identify the phosphorylation sites of DDHD1. The search parameters in MASCOT were the following: number of missed and/or nonspecific cleavages permitted: 1; list of all fixed modifications (including residue specificity) considered: None; list of all variable modifications (including residue specificity) considered: Phospho (ST); Mass tolerance for precursor ions: ±0.9 to 1.4 Da; Mass tolerance for fragment ions: ±1.3 to 2.0 Da. To prevent the hydrolysis of phosphate groups, all solutions used in this process were adjusted to below pH 8.3.

### Laemmli SDS-PAGE and Phos-tag SDS-PAGE

Total proteins were extracted using RIPA buffer (10 mM Tris-HCl pH 7.4, 150 mM NaCl, 1 mM EDTA, 1% (w/v) NP-40, 0.1% (w/v) SDS, and 0.5% sodium deoxycholate) supplemented with protease inhibitors. Regarding Phos-tag PAGE, 1 × 10^7^ cells were also lysed using the same RIPA buffer without EDTA. Laemmli SDS-PAGE was performed with 10% polyacrylamide gels. Proteins were transferred to nitrocellulose membranes (Wako) using a semidry blotting apparatus (model AE-6675 or AE-6677, ATTO). Zn^2+^-Phos-tag SDS-PAGE was performed using the Tris-AcOH (acetic acid) system with a 3% polyacrylamide gel strengthened with 0.5% agarose H (Wako) using a procedure modified from a previously published protocol (29, 38). We only slightly succeeded in separating the phosphorylated forms of DDHD1 only using this Tris-AcOH system with a low-concentration polyacrylamide gel because of the high-molecular mass of the protein (38). After electrophoresis, Phos-tag acrylamide gels were washed twice with transfer buffer (25 mm Tris, 192 mm glycine, 20% methanol) containing 10 mm EDTA for 10 min with gentle shaking and then with transfer buffer without EDTA for 10 min according to the manufacturer’s protocol. Proteins were transferred to nitrocellulose membranes. In both cases, membranes were blocked with ImmunoBlock (DS Pharma) and probed with anti-FLAG M2 (Sigma), followed by the horseradish-peroxidase-conjugated secondary antibody. Immunodetection was performed with Clarity Western ECL Substrate (Bio-Rad) or Western BLoT Ultra Sensitive HRP Substrate (Takara) by Amersham Imager 680 (GE Healthcare).

### Analysis of PLA1 activity

PLA1 activity was measured using the EnzChek Phospholipase A1 assay kit (Thermo Fisher Scientific) according to the manufacturer’s protocol. PED-A1 is a glycerophosphoethanolamine with BODIPY FL pentanoic acid at the *sn*-1 position and a dinitrophenyl quencher-modified head group. The cleavage of the BODIPY FL-labeled acid at the *sn*-1 position by PLA1 reduces quenching efficiency and increases fluorescence emission detected at approximately 515 nm. Mixed phospholipid micelles were prepared as PED-A1/dioleoylphosphatidylcholine (DOPC)/dioleoylphosphatidylglycerol (DOPG) (9:45:45, by mol) in 50 mM Tris-HCl (pH 7.4), 140 mM NaCl, and 2 mM CaCl_2_. The substrate concentration in the micelles was 36.7 μM (3.33 μM PED-A1). Reactions were initiated by the addition of 50 ng of purified FLAG-DDHD1 and its mutants and were conducted at room temperature for 10 to 30 min. The fluorescent cleavage product was measured (450–490 nm excitation, 515 nm emission) by a Powerscan HT multidetection microplate reader (DS Pharma).

We confirmed that activity monitored by 515-nm emission was dependent on the amount of FLAG-DDHD1 and the incubation period. In addition, the incubation with the inactive mutant, in which the serine 537 residue involved in PLA1 activity was substituted to alanine, did not increase the 515-nm emission (11).

### Preparation of fluorescent anti-ALFA nanobody

The anti-ALFA nanobody cDNA containing a poly (Asp)_6_ tag at the C-terminus was synthesized (Integrated DNA Technologies), *in vitro* assembled with cDNA of cfSGFP2 (39) or tdTomato (40) as a N-terminal tag using NEBuilder (NEB), and inserted into an Eco53kI site of pColdII (Takara). The poly Asp tag, which is a variant of the SEP tag (41), was used to increase extraction efficiency. Protein extraction was essentially carried out according to the manufacturer’s protocol. Briefly, the recombinant protein was expressed in OverExpress C41 cells (Sigma) at 15 °C for 48 h and extracted from the cells in extraction buffer (0.5% Triton X-100, 50 mM NaCl, 100 mM Tris-HCl, pH 7.5, and 8 mM imidazole) with sonication. The cleared lysate was applied onto Talon resin (Takara), which was then washed with high-salt buffer (500 mM NaCl, 100 mM Tris-HCl, pH 7.5, and 8 mM imidazole). The recombinant protein was eluted with elution buffer (200 mM imidazole, 150 mM NaCl, and 100 mM HEPES, pH 7.5) and used at 5 to 10 μg/ml for immunostaining.

### Immunofluorescence (IF)

Cells transfected with plasmids plated onto retronectin (Takara)-coated cover glasses (D263M, Schott) were fixed with glyoxal fixative (42) and blocked using 10% normal goat serum in IF buffer (0.25 M ammonium chloride, 0.25 M potassium acetate, 10 mM EDTA, 1 mM EGTA, 50 mM Tris-HCl, pH 7.5, and 0.1% Triton X-100) for 30 min. They were then incubated with anti-FLAG antibody or cfSGFP2-labeled anti-ALFA nanobody in 2% BSA-containing IF buffer for 30 min and further incubated with Alexa-568-labeled anti-rabbit antibody (ThermoFisher). Fluorescence images were acquired using a 100 × oil-immersion objective (NA 1.49) or 60 × water-immersion objective (NA 1.27) by confocal microscopy with A1R (NIKON) as described previously (43).

### *In vitro* kinase assay

Purified FLAG-tagged DDHD1 (WT or mutant) was resuspended in kinase assay buffer (40 mM Tris-HCl, pH 7.4, and 20 mM MgCl_2_) containing 1 μM ATP, 50 μM DTT, and 0.1 to 1 mg/ml of BSA together with either kinases, kinase complexes, or kinase mutants. Reaction mixtures were incubated at 25 °C for 150, 180, or 360 min. The reaction was stopped by the addition of Laemmli sample buffer, followed by Phos-tag SDS–PAGE.

## Data availability

The MS/MS data were deposited into the ProteomeXchange Consortium *via* the jPOST partner repository with the data set identifier PXD023208.

## Supporting information

This article contains supporting information.

## Conflict of interest

The authors declare that they have no conflict of interest regarding the content of this article.

## Supporting information

Suppplemental Figures S1–S7 and Table S1
