# Supplementary material for: Phosphorylation of human phospholipase A1 DDHD1 at newly identified phosphosites affects its subcellular localization
Source: J Biol Chem. 2021 Jun 3;297(1):100851. doi: 10.1016/j.jbc.2021.100851 (PMC8234217; doi:10.1016/j.jbc.2021.100851)
Supplement: Suppplemental Figures S1–S7 and Table S1 [file mmc1.pdf]

Phosphorylation of human phospholipase A1 DDHD1 at newly identified phosphosites affects its subcellular localization

Naoki Matsumoto [‡], Yoko Nemoto-Sasaki [‡], Saori Oka [‡], Seisuke Arai[§], Ikuo Wada[§], and Atsushi Yamashita [‡] *

[‡] Faculty of Pharma-Science, Teikyo University, 2-11-1 Kaga, Itabashi-Ku, Tokyo 173-8605, Japan, and [§]Department of Cell Science, Institute of Biomedical Sciences, Fukushima Medical University School of Medicine, Fukushima City, Fukushima 960-1295, Japan

*To whom correspondence should be addressed: Atsushi Yamashita, Faculty of Pharma-Science, Teikyo University, 2-11-1 Kaga, Itabashi-Ku, Tokyo 173-8605, Japan; E-mail address: ayamashi@pharm.teikyo-u.ac.jp; Tel.: +81-3-3964-8149

Running Title: Multi-site phosphorylation of human DDHD1 phospholipase A1

File Name: Supporting Information

Description: 7 Supporting Figures and one Supporting Table

Figure S1. Schematic diagram showing structures of wild-type and amino acid-substituted forms of human DDHD1 and protein kinases/their activators employed in the present study. The positions of the substitutions of serine/threonine to alanine/glutamic acid in DDHD1 are shown in magenta or green, respectively. Schematic diagrams of protein kinases and their activators are also depicted. For kinase-inactivated CK2α1, amino acid substitutions are shown in yellow. FLAG or ALFA epitope-tagging in the amino terminus is depicted as black. The FLAG-tag in the carboxy terminus of cyclin A2 is also black.

Figure S2. The spectrum of phosphopeptides of DDHD1.
The phosphopeptides were purified as described in the legend of Figure 2 and were analyzed by MALDI-TOF MS/MS. MS/MS spectra of mono- or di-phosphorylated peptide at Ser8 (A), at Ser11 (B), at Ser104 and Ser139 (C), at Ser130 (D), at Ser139 (E), at Ser332 (F), at Ser723 (G), at Ser738 (H), and at Ser806 (I) are depicted. The sequences of identified peptides are shown above the mass spectra.

Figure S3. Phosphorylation state of human DDHD1 in other human cell lines.
Wild-type FLAG-DDHD1 (WT), single (S8A, S11A, and S727A), and multiple (S8/11/727A) Ser-to-Ala substitution mutants were expressed in HeLa (A), HepG2 (B), or PANC1 cells (C), and electrophoretic mobility shifts in WT and Ala substitution mutants of human DDHD1 by phosphorylation are shown. WT and Ala substitution mutant-expressing human cells were cultured in growth medium containing 10% FBS. Cell extracts were analyzed by Zn^{2+} Phos-tag SDS-PAGE (upper panels) or normal SDS-PAGE (lower panels) following immunoblot analysis (IB). The R_f value of 1.0 is defined as the position of bromphenol blue dye.

Figure S4. DDHD1 has minor phosphorylation sites besides Ser8, Ser11, Ser723, and Ser727.
Wild-type FLAG-DDHD1 (WT), single (S8A, S11A, S723A, and S727A), and multiple (S8/11/723/727A) Ser-to-Ala substitution mutants were expressed in HEK293 cells, and electrophoretic mobility shifts in WT and Ala substitution mutants of human DDHD1 by the inhibition of protein phosphatases are shown. WT and Ala substitution mutant-expressing HEK293 cells were cultured in the presence (lanes 1-6) or absence (lane 7) of 1 μM okadaic acid for 4 hours in growth medium containing 10% FBS. Cell extracts were analyzed by Zn^{2+} Phos-tag SDS-PAGE following immunoblot analysis (IB). The R_f value of 1.0 is defined as the position of bromphenol blue dye.

Figure S5. Assay of PLA1 activity is accurate and reliable, but alanine-substitutions of

DDHD1 (phosphorylation-resistant forms) do not greatly alter PLA1 activity.

The accuracy and reliability of PLA1 assay were confirmed. The PLA1 activity of purified recombinant FLAG-DDHD1 (WT) was measured using a fluorescent substrate (PED-A1). (A) The PLA1 activity of five doses of WT quantified by bicinchoninic acid assay (12.5, 25, 50, 100, or 200 ng) was measured in triplicate. Each individual point is represented as a small circle. (B) The PLA1 activity of 50 ng of WT was measured after 8, 10, 20, or 30 min in triplicate. Each individual point is represented as a small circle, triangle, or square (green, red, or black linear approximations, respectively). (C) The PLA1 activity of purified recombinant WT, S11A, S727A, and S11/727A was measured. Fifty nanograms of purified WT and mutants was used after quantification by the bicinchoninic acid assay. Results are expressed as the mean ± SD of an experiment performed in triplicate. Each individual point is also represented as a small circle.

Figure S6. Knock-down of GSK3β or CDK1 does not alter the phosphorylation of DDHD1 in HEK293 cells.

(A) The double-strand oligonucleotides corresponding to control, GSK3β, or CDK1 were inserted into a miRNA expression vector (pcDNA 6.2-GW/EmGFP-miR). (B) The miRNA expression vectors for GSK3β or CDK1 down-regulated GSK3β or CDK1, but not DDHD1 (FLAG) based on Western blotting analysis. (C) The miRNA expression vectors for GSK3β or CDK1 did not alter the phosphorylation of DDHD1 in HEK 293 cells based on Zn^{2+}Phos-tag SDS-PAGE.

Figure S7. *In vitro* kinase assay of DDHD1 by GSK3β (A), ERK2 (B), p38α (C), and GSK3α (C).

(A) Purified recombinant wild-type FLAG-DDHD1 (WT, lanes 3-5), its dephosphorylated form (λPP-treated WT, lanes 6-8), or TBS (none, lanes 1 and 2) was incubated in combination with purified FLAG-tagged GSK3β1 or its splicing variant, GSK3β2, at 25°C for 180 min. (B) Recombinant DDHD1 (WT, lanes 2 and 3), its dephosphorylated form (λPP-treated WT, lanes 4 and 5), or TBS (none, lane 1) was incubated in combination with purified FLAG-tagged ERK2 at 25°C for 180 min. (C) Recombinant DDHD1 (WT, lanes 3-5), its dephosphorylated form (λPP-treated WT, lanes 6-8), or TBS (none, lanes 1 and 2) was incubated in combination with purified FLAG-tagged p38α or GSK3α at 25°C for 180 min. The incubated mixtures containing equivalent amounts of DDHD1 were analyzed by Zn^{2+} Phos-tag SDS-PAGE following immunoblot analysis using anti-FLAG M2 antibody. The R_f value of 1.0 is defined as the position of bromphenol blue dye.

Table S1. NetPhos 3.1 prediction results

Posphorylation sites	Context	Score	Kinase
Ser8	PGRGSPRSP	0.997	unsp
Ser8	PGRGSPRSP	0.587	cdk5
Ser8	PGRGSPRSP	0.526	GSK3
Ser8	PGRGSPRSP	0.525	p38MAPK
Ser11	GSPRSPEHN	0.996	unsp
Ser11	GSPRSPEHN	0.610	cdk5
Ser11	GSPRSPEHN	0.585	p38MAPK
Ser11	GSPRSPEHN	0.511	GSK3
Ser104	LRYYSEGES	0.995	unsp
Ser104	LRYYSEGES	0.525	PKG
Ser130	VPTNSGGGG	0.549	CKI
Ser139	ATGGSPGER	0.980	unsp
Ser139	ATGGSPGER	0.537	cdc2
Ser139	ATGGSPGER	0.530	cdk5
Ser139	ATGGSPGER	0.512	GSK3
Ser332	EVSKSIDGK	0.971	unsp
Ser723	STIPSPVTS	0.840	unsp
Ser723	STIPSPVTS	0.631	cdk5
Ser723	STIPSPVTS	0.524	GSK3
Ser723	STIPSPVTS	0.504	p38MAP
Thr726	PSPVTSPVL	-	-
Ser727	SPVTSPVLS	0.919	unsp
Ser727	SPVTSPVLS	0.709	cdk5
Ser727	SPVTSPVLS	0.546	p38MAPK
Ser727	SPVTSPVLS	0.532	GSK3
Ser738	HYGESITNI	0.538	PKC
Ser738	HYGESITNI	0.515	CKI
Ser806	PHSSSGFLD	0.536	cdc2

Context: the sequence context of the residue, shown as a 9-residue subsequence centered on the residue
Score: the prediction score (a value in the range [0.000-1.000]; the scores above 0.500 indicate positive predictions)
Kinase: the active kinase or the string "unsp" for non-specific prediction

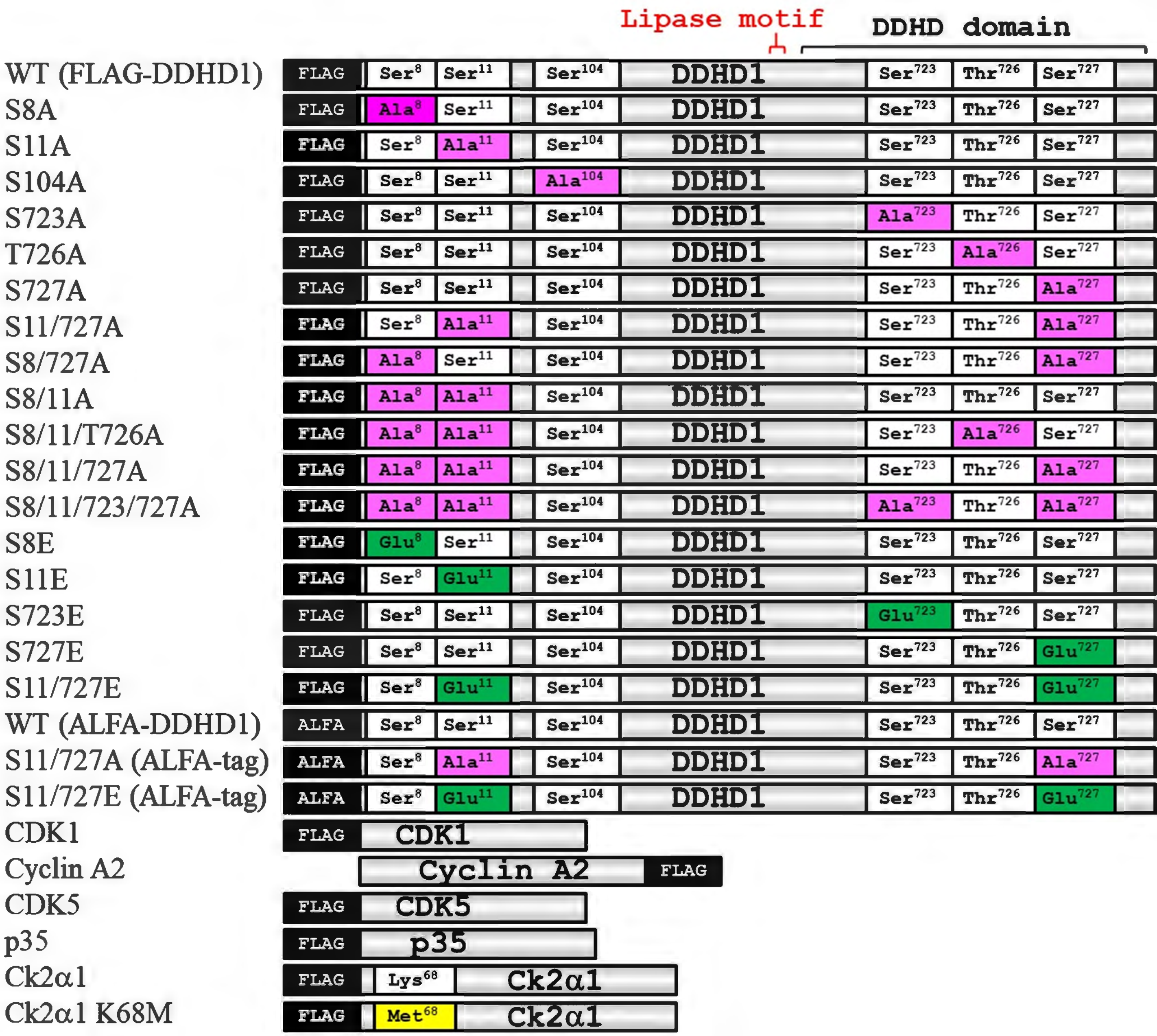

Matsumoto *et al.*

Figure S1

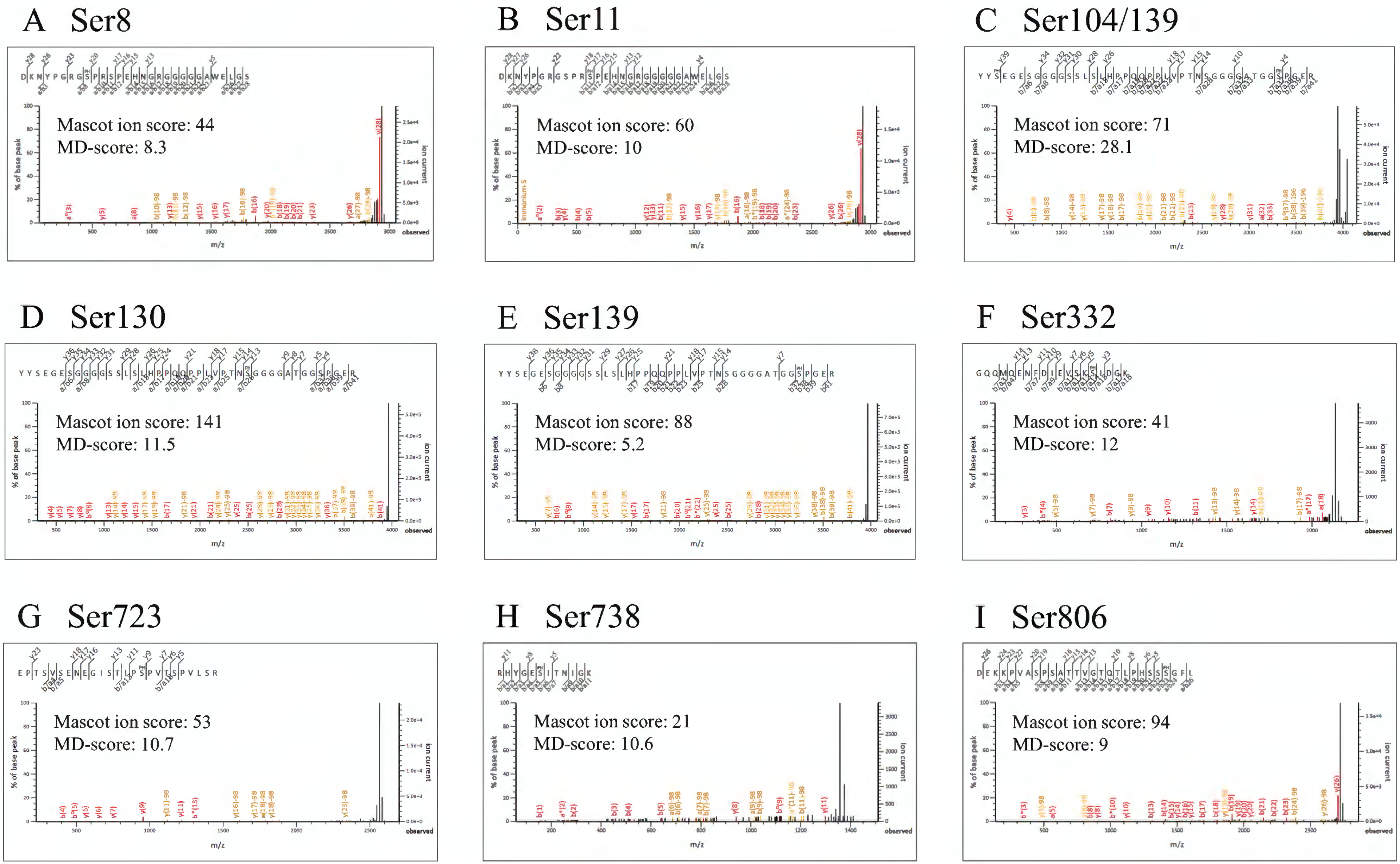

Matsumoto *et al.*

Figure S2

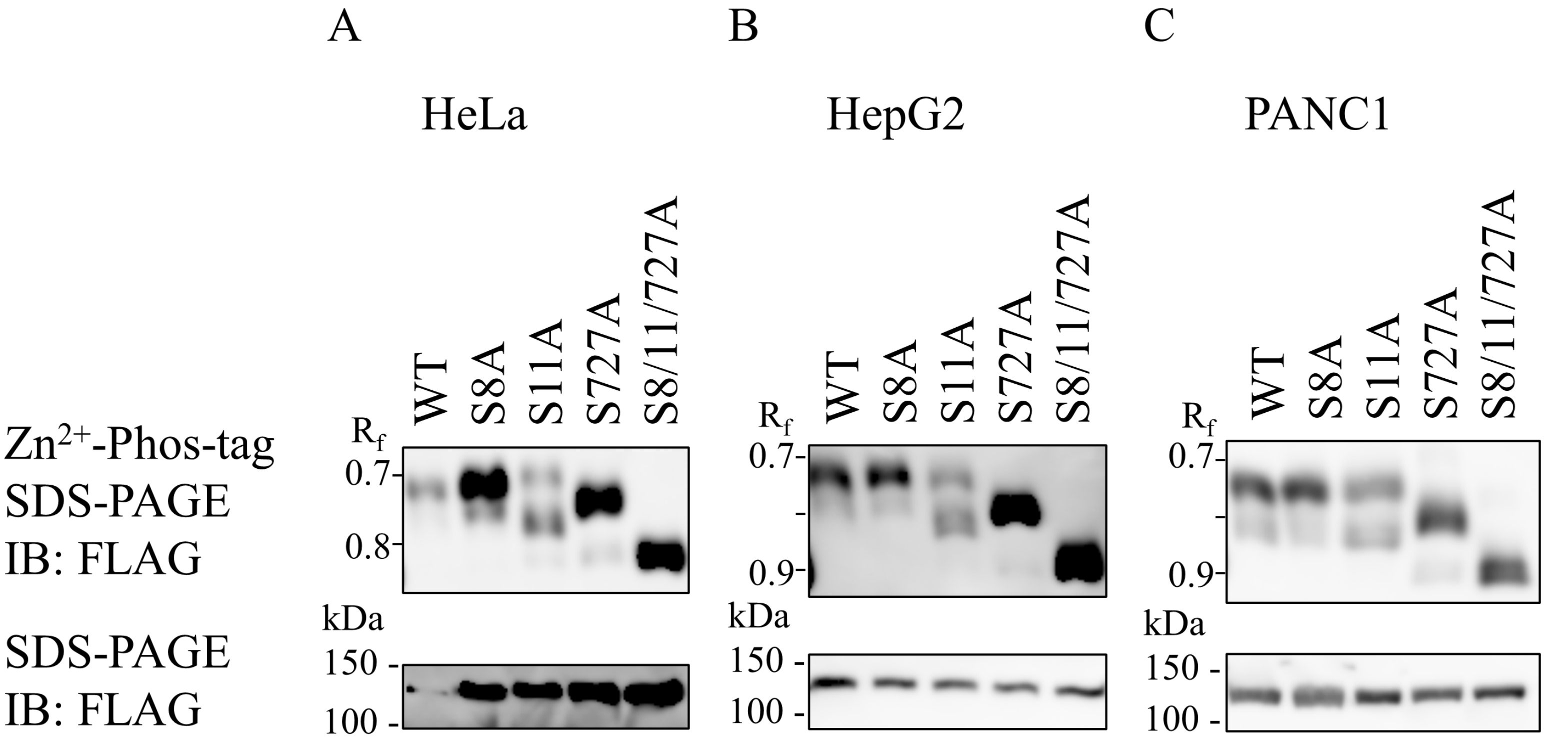

Matsumoto *et al.*

Figure S3

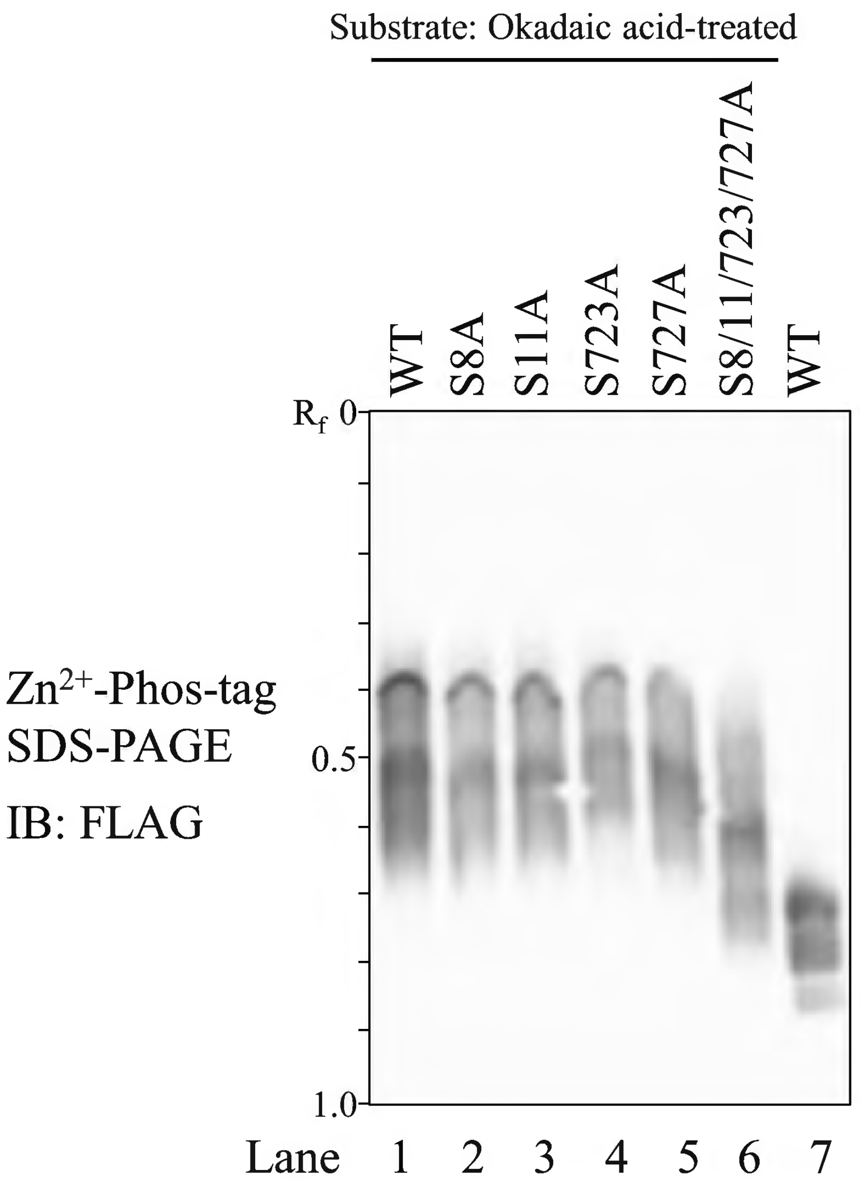

Matsumoto *et al.*

Figure S4

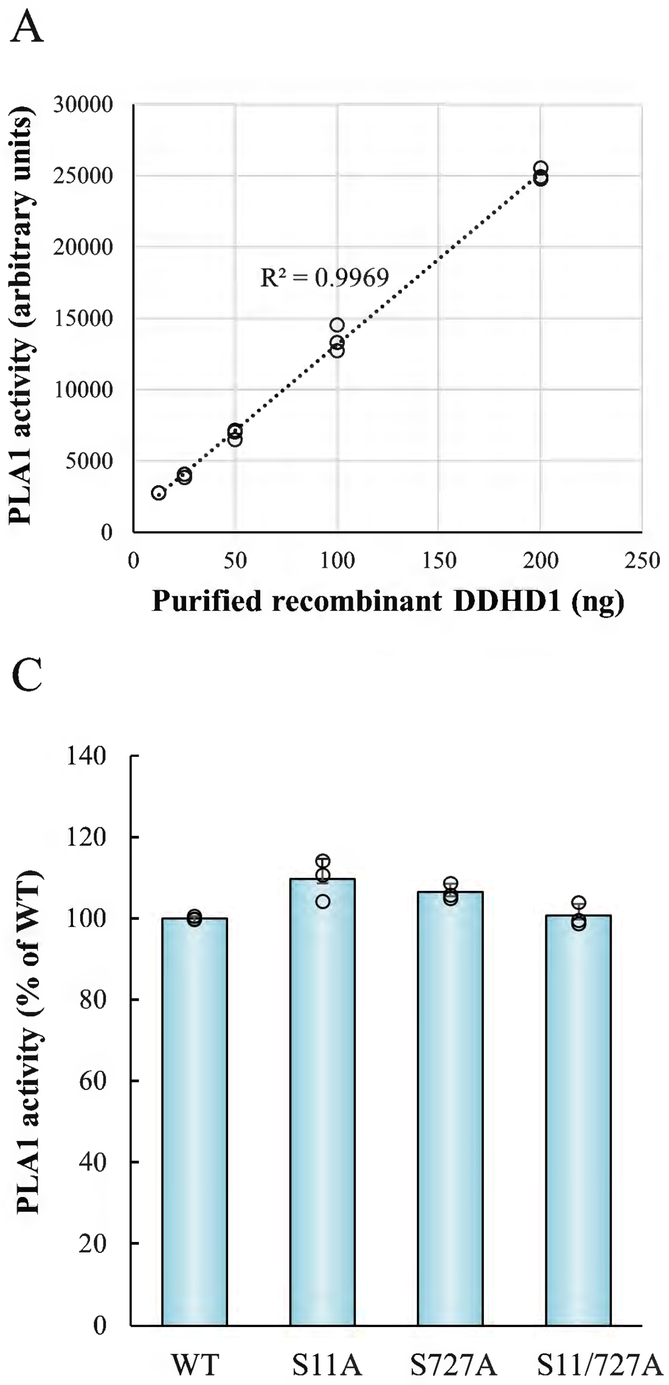

Matsumoto *et al.*

Figure S5

A

Primer set		Primer sequence (5' to 3')
miR-neg control	Forward	TGCTGAAATGTACTGCGCGTGGAGACGTTTTGGCCACTGACTGACGTCTCCACGCAGTACATTT
	Reverse	CCTGAAATGTACTGCGTGGAGACGTCAGTCAGTGGCCAAAACGTCTCCACGCGCAGTACATTTC
GSK3β miRNA1	Forward	TGCTGTGCTGAGTGACACTCAAGTAAGTTTTGGCCACTGACTGACTTACTTGAGTCACTCAGCA
	Reverse	CCTGTGCTGAGTGACTCAAGTAAGTCAGTCAGTGGCCAAAACTTACTTGAGTGTCACTCAGCAC
GSK3β miRNA5	Forward	TGCTGAACGCAATCGGACTATGTTACGTTTTGGCCACTGACTGACGTAACATACCGATTGCGTT
	Reverse	CCTGAACGCAATCGGTATGTTACGTCAGTCAGTGGCCAAAACGTAACATAGTCCGATTGCGTTC
CDK1 miRNA1	Forward	TGCTGTTACAGTGAGACCTACACACAGTTTTGGCCACTGACTGACTGTGTGTATCTCACTGTAA
	Reverse	CCTGTTACAGTGAGATACACACAGTCAGTCAGTGGCCAAAACTGTGTGTAGGTCTCACTGTAAC
CDK1 miRNA4	Forward	TGCTGTAACCTGGAATCCTGCATAAGGTTTTGGCCACTGACTGACCTTATGCAATTCCAGGTTA
	Reverse	CCTGTAACCTGGAATTGCATAAGGTCAGTCAGTGGCCAAAACCTTATGCAGGATTCCAGGTTAC
CDK1 miRNA5	Forward	TGCTGTGTACCAGAGTGTTACTACCTGTTTTGGCCACTGACTGACAGGTAGTAACTCTGGTACA
	Reverse	CCTGTGTACCAGAGTTACTACCTGTCAGTCAGTGGCCAAAACAGGTAGTAACACTCTGGTACAC

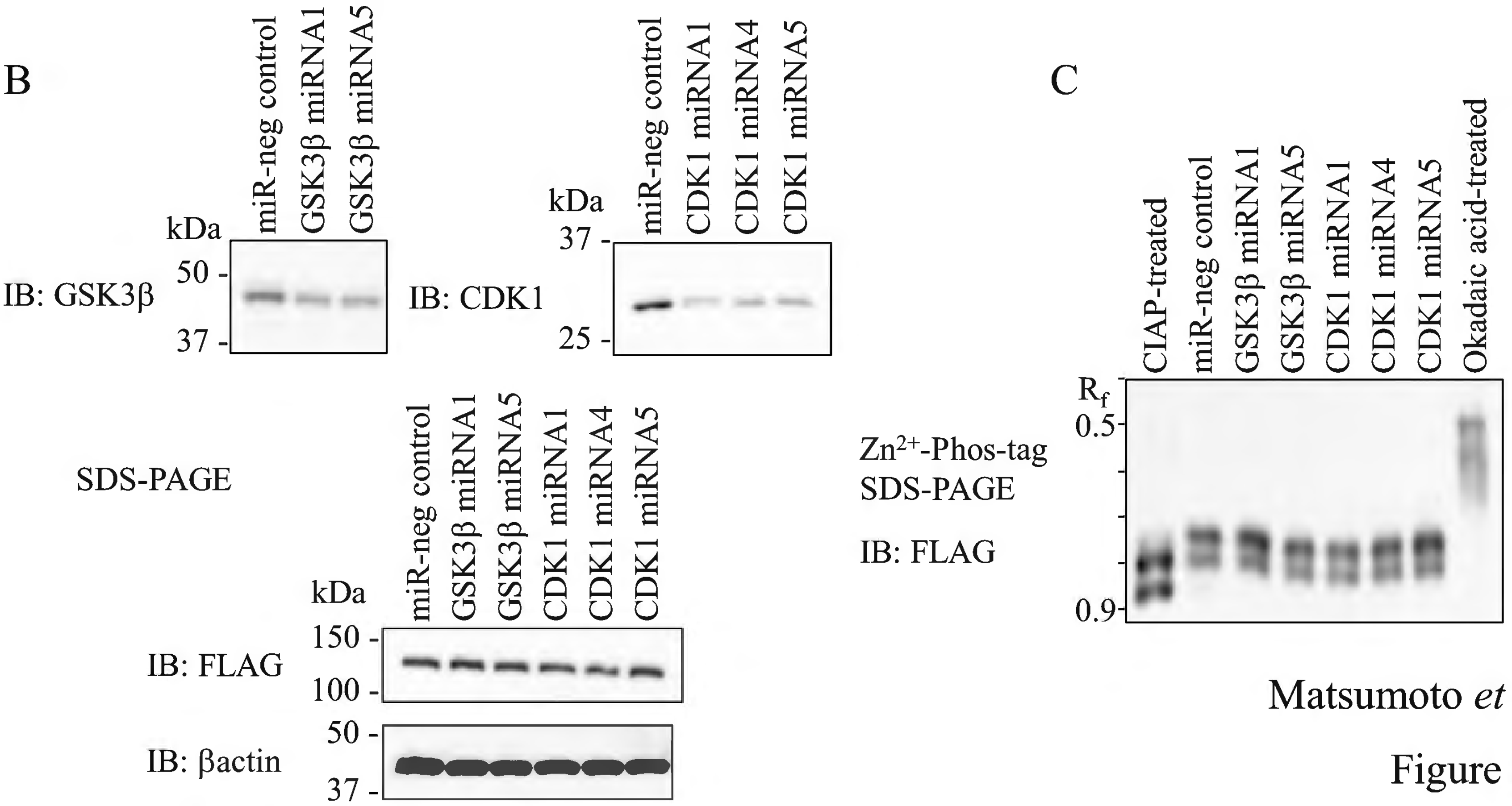

Matsumoto *et al.*

Figure S6

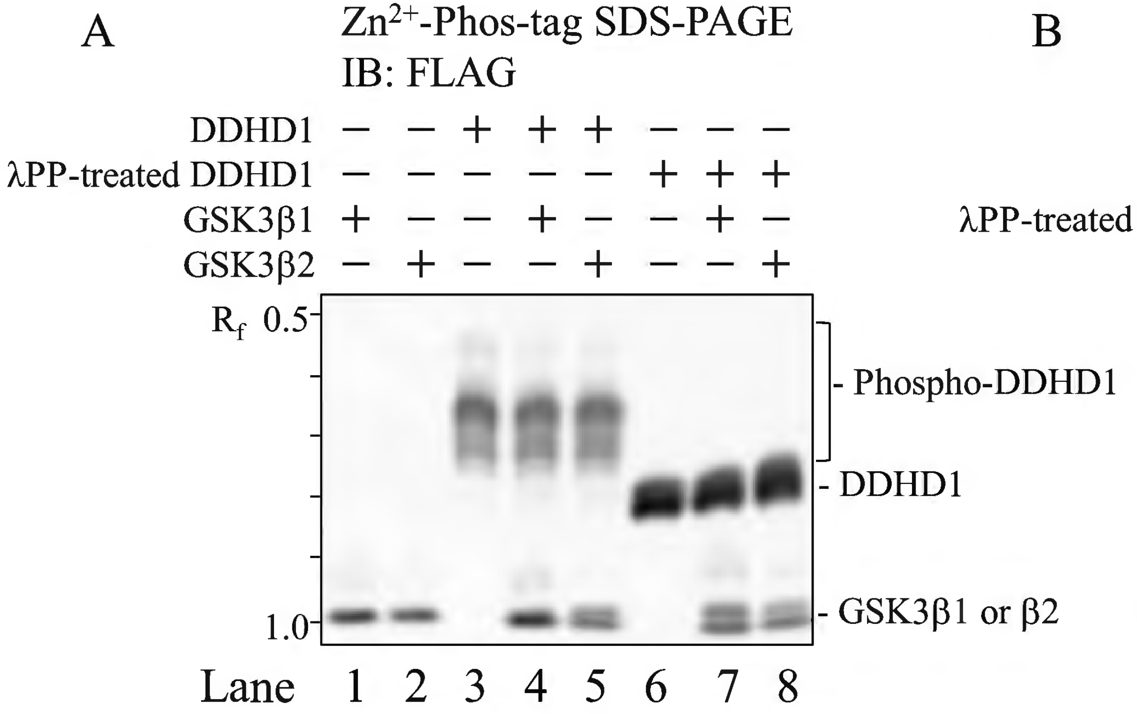

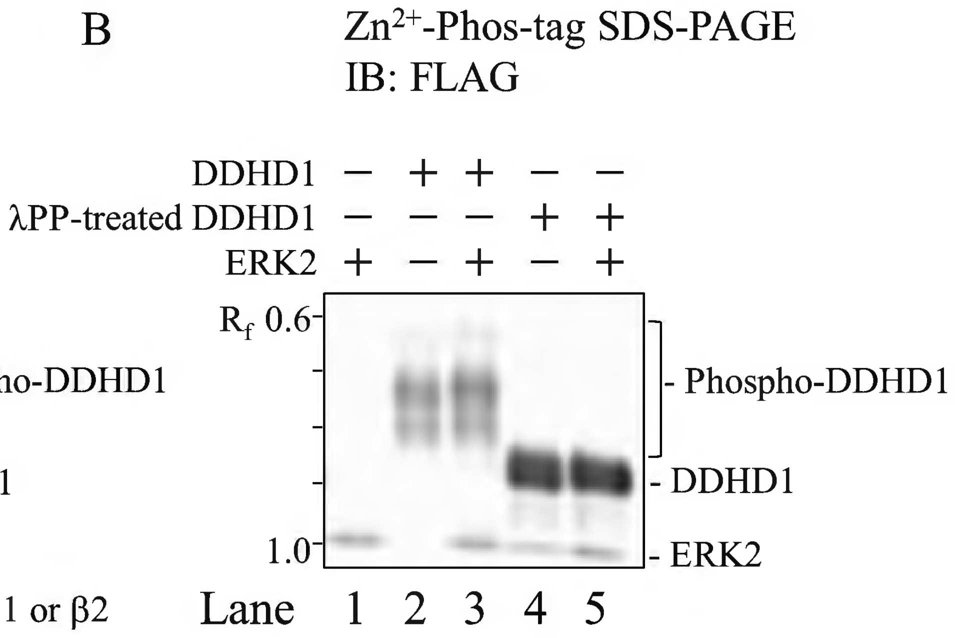

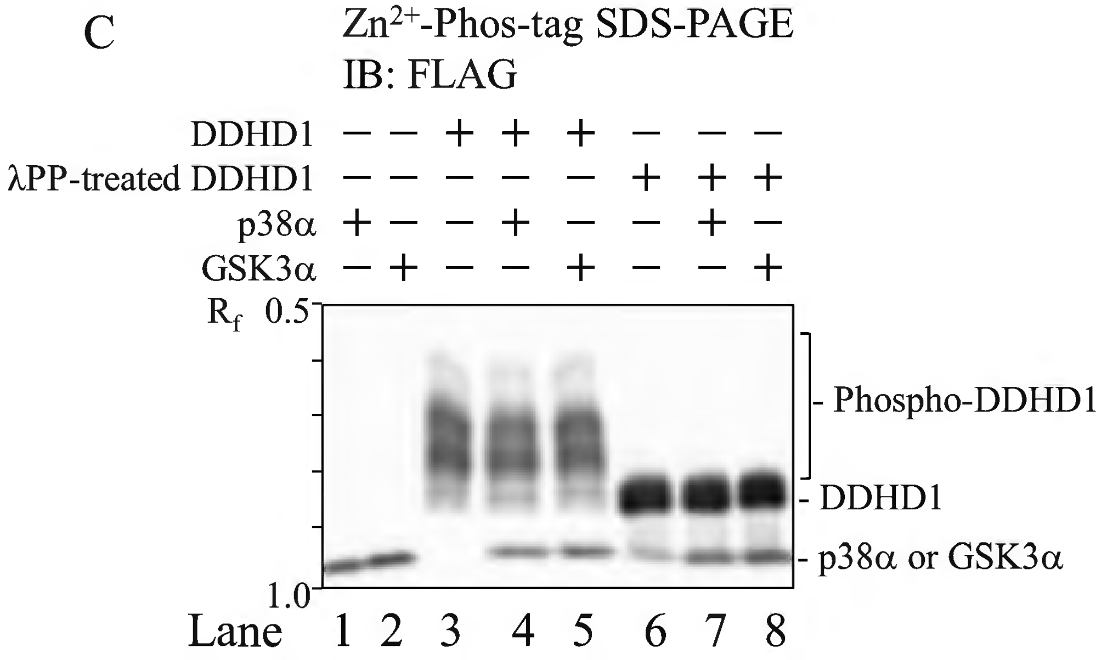

Matsumoto *et al.*

Figure S7
